# Large-scale targeted sequencing identifies risk genes for neurodevelopmental disorders

**DOI:** 10.1038/s41467-020-18723-y

**Published:** 2020-10-01

**Authors:** Tianyun Wang, Kendra Hoekzema, Davide Vecchio, Huidan Wu, Arvis Sulovari, Bradley P. Coe, Madelyn A. Gillentine, Amy B. Wilfert, Luis A. Perez-Jurado, Malin Kvarnung, Yoeri Sleyp, Rachel K. Earl, Jill A. Rosenfeld, Madeleine R. Geisheker, Lin Han, Bing Du, Chris Barnett, Elizabeth Thompson, Marie Shaw, Renee Carroll, Kathryn Friend, Rachael Catford, Elizabeth E. Palmer, Xiaobing Zou, Jianjun Ou, Honghui Li, Hui Guo, Jennifer Gerdts, Emanuela Avola, Giuseppe Calabrese, Maurizio Elia, Donatella Greco, Anna Lindstrand, Ann Nordgren, Britt-Marie Anderlid, Geert Vandeweyer, Anke Van Dijck, Nathalie Van der Aa, Brooke McKenna, Miroslava Hancarova, Sarka Bendova, Marketa Havlovicova, Giovanni Malerba, Bernardo Dalla Bernardina, Pierandrea Muglia, Arie van Haeringen, Mariette J. V. Hoffer, Barbara Franke, Gerarda Cappuccio, Martin Delatycki, Paul J. Lockhart, Melanie A. Manning, Pengfei Liu, Ingrid E. Scheffer, Nicola Brunetti-Pierri, Nanda Rommelse, David G. Amaral, Gijs W. E. Santen, Elisabetta Trabetti, Zdeněk Sedláček, Jacob J. Michaelson, Karen Pierce, Eric Courchesne, R. Frank Kooy, The SPARK Consortium, John Acampado, Andrea J. Ace, Alpha Amatya, Irina Astrovskaya, Asif Bashar, Elizabeth Brooks, Martin E. Butler, Lindsey A. Cartner, Wubin Chin, Wendy K. Chung, Amy M. Daniels, Pamela Feliciano, Chris Fleisch, Swami Ganesan, William Jensen, Alex E. Lash, Richard Marini, Vincent J. Myers, Eirene O’Connor, Chris Rigby, Beverly E. Robertson, Neelay Shah, Swapnil Shah, Emily Singer, LeeAnne G. Snyder, Alexandra N. Stephens, Jennifer Tjernagel, Brianna M. Vernoia, Natalia Volfovsky, Loran Casey White, Alexander Hsieh, Yufeng Shen, Xueya Zhou, Tychele N. Turner, Ethan Bahl, Taylor R. Thomas, Leo Brueggeman, Tanner Koomar, Jacob J. Michaelson, Brian J. O’Roak, Rebecca A. Barnard, Richard A. Gibbs, Donna Muzny, Aniko Sabo, Kelli L. Baalman Ahmed, Evan E. Eichler, Matthew Siegel, Leonard Abbeduto, David G. Amaral, Brittani A. Hilscher, Deana Li, Kaitlin Smith, Samantha Thompson, Charles Albright, Eric M. Butter, Sara Eldred, Nathan Hanna, Mark Jones, Daniel Lee Coury, Jessica Scherr, Taylor Pifher, Erin Roby, Brandy Dennis, Lorrin Higgins, Melissa Brown, Michael Alessandri, Anibal Gutierrez, Melissa N. Hale, Lynette M. Herbert, Hoa Lam Schneider, Giancarla David, Robert D. Annett, Dustin E. Sarver, Ivette Arriaga, Alexies Camba, Amanda C. Gulsrud, Monica Haley, James T. McCracken, Sophia Sandhu, Maira Tafolla, Wha S. Yang, Laura A. Carpenter, Catherine C. Bradley, Frampton Gwynette, Patricia Manning, Rebecca Shaffer, Carrie Thomas, Raphael A. Bernier, Emily A. Fox, Jennifer A. Gerdts, Micah Pepper, Theodore Ho, Daniel Cho, Joseph Piven, Holly Lechniak, Latha V. Soorya, Rachel Gordon, Allison Wainer, Lisa Yeh, Cesar Ochoa-Lubinoff, Nicole Russo, Elizabeth Berry-Kravis, Stephanie Booker, Craig A. Erickson, Lisa M. Prock, Katherine G. Pawlowski, Emily T. Matthews, Stephanie J. Brewster, Margaret A. Hojlo, Evi Abada, Elena Lamarche, Tianyun Wang, Shwetha C. Murali, William T. Harvey, Hannah E. Kaplan, Karen L. Pierce, Lindsey DeMarco, Susannah Horner, Juhi Pandey, Samantha Plate, Mustafa Sahin, Katherine D. Riley, Erin Carmody, Julia Constantini, Amy Esler, Ali Fatemi, Hanna Hutter, Rebecca J. Landa, Alexander P. McKenzie, Jason Neely, Vini Singh, Bonnie Van Metre, Ericka L. Wodka, Eric J. Fombonne, Lark Y. Huang-Storms, Lillian D. Pacheco, Sarah A. Mastel, Leigh A. Coppola, Sunday Francis, Andrea Jarrett, Suma Jacob, Natasha Lillie, Jaclyn Gunderson, Dalia Istephanous, Laura Simon, Ori Wasserberg, Angela L. Rachubinski, Cordelia R. Rosenberg, Stephen M. Kanne, Amanda D. Shocklee, Nicole Takahashi, Shelby L. Bridwell, Rebecca L. Klimczac, Melissa A. Mahurin, Hannah E. Cotrell, Cortaiga A. Grant, Samantha G. Hunter, Christa Lese Martin, Cora M. Taylor, Lauren K. Walsh, Katherine A. Dent, Andrew Mason, Anthony Sziklay, Christopher J. Smith, Magnus Nordenskjöld, Corrado Romano, Hilde Peeters, Raphael A. Bernier, Jozef Gecz, Kun Xia, Evan E. Eichler

**Affiliations:** 1 Department of Genome Sciences, University of Washington, Seattle, WA USA; 2 Rare Disease and Medical Genetics, Academic Department of Pediatrics, Bambino Gesù Children’s Hospital, Rome, Italy; 3 Genetics and Rare Diseases Research Division, Bambino Gesù Children’s Hospital, Rome, Italy; 4 Center for Medical Genetics & Hunan Provincial Key Laboratory of Medical Genetics, School of Life Sciences, Central South University, Changsha, Hunan China; 5 Paediatric and Reproductive Genetics unit, Women’s and Children’s Hospital, Adelaide, SA Australia; 6 South Australian Health and Medical Research Institute, Adelaide, SA Australia; 7 Genetics Unit, Universitat Pompeu Fabra, Hospital del Mar Research Institute (IMIM) and CIBERER, Barcelona, Spain; 8 Department of Molecular Medicine and Surgery, Center for Molecular Medicine, Karolinska Institutet, Stockholm, Sweden; 9 Department of Clinical Genetics, Karolinska University Hospital, Stockholm, Sweden; 10 Centre for Human Genetics, KU Leuven and Leuven Autism Research (LAuRes), Leuven, Belgium; 11 Department of Psychiatry and Behavioral Sciences, University of Washington, Seattle, WA USA; 12 Department of Molecular & Human Genetics, Baylor College of Medicine, Houston, TX USA; 13 Baylor Genetics, Houston, TX USA; 14 Adelaide Medical School and the Robinson Research Institute, the University of Adelaide, Adelaide, SA Australia; 15 Genetics and Molecular Pathology, SA Pathology, Adelaide, SA Australia; 16 Genetics of Learning Disability Service, Hunter New England Health Service, Waratah, NSW Australia; 17 School of Women’s and Children’s Health, University of New South Wales, Randwick, NSW Australia; 18 Children Development Behavior Center, The Third Affiliated Hospital, Sun Yat-Sen University, Guangzhou, Guangdong, China; 19 Mental Health Institute of the Second Xiangya Hospital, Central South University, Changsha, China; 20 Key Laboratory of Developmental Disorders in Children, Liuzhou Maternity and Child Healthcare Hospital, Liuzhou, China; 21 Oasi Research Institute-IRCCS, Troina, Italy; 22 Department of Medical Genetics, University of Antwerp, Antwerp, Belgium; 23 Department of Psychology, Emory University, Atlanta, GA USA; 24 Department of Biology and Medical Genetics, Charles University 2nd Faculty of Medicine and University Hospital Motol, Prague, Czech Republic; 25 Department of Neurosciences, Biomedicine and Movement Sciences, University of Verona, Verona, Italy; 26 Child Neuropsychiatry Unit, AOUI, Verona, Italy; 27 UCB Pharma, Bruxelles, Belgium; 28 Department of Clinical Genetics, Leiden University Medical Center (LUMC), Leiden, Netherlands; 29 Department of Human Genetics, Donders Institute for Brain, Cognition and Behaviour, Radboud University Medical Center, Nijmegen, Netherlands; 30 Department of Psychiatry, Donders Institute for Brain, Cognition and Behaviour, Radboud University Medical Center, Nijmegen, Netherlands; 31 Department of Translational Medicine, Federico II University, Naples, Italy; 32 Telethon Institute of Genetics and Medicine, Pozzuoli, Naples, Italy; 33 Murdoch Children’s Research Institute, Melbourne, Australia; 34 Department of Paediatrics, University of Melbourne, Parkville, VIC Australia; 35 Division of Medical Genetics, Department of Pediatrics, Stanford University, Stanford, CA USA; 36 Department of Pathology, Stanford University, Stanford, CA USA; 37 Department of Paediatrics, University of Melbourne, Royal Children’s Hospital, Melbourne, VIC Australia; 38 Department of Medicine, University of Melbourne, Austin Health, Melbourne, Australia; 39 The Florey Institute of Neuroscience and Mental Health, Parkville, VIC Australia; 40 Karakter Child and Adolescent Psychiatry Center, Nijmegen, Netherlands; 41 Department of Psychiatry and Behavioral Sciences and the MIND Institute, University of California, Davis, Sacramento, CA USA; 42 Department of Psychiatry, University of Iowa Carver College of Medicine, Iowa City, IA USA; 43 Department of Neurosciences, UC San Diego Autism Center, School of Medicine, University of California San Diego, La Jolla, CA USA; 44 CAS Center for Excellence in Brain Science and Intelligences Technology (CEBSIT), Chinese Academy of Sciences, Shanghai, China; 45 Howard Hughes Medical Institute, University of Washington, Seattle, WA USA; 46 Simons Foundation, New York, NY USA; 47 Columbia University, New York, NY USA; 48 Washington University School of Medicine, St. Louis, MO USA; 49 University of Iowa Carver College of Medicine, Iowa City, IA USA; 50 Oregon Health & Science University, Portland, OR USA; 51 Baylor College of Medicine, Houston, TX USA; 52 University of Washington School of Medicine & Howard Hughes Medical Institute, Seattle, WA USA; 53 Maine Medical Center Research Institute, Portland, OR USA; 54 University of California, Davis, Sacramento, CA USA; 55 Nationwide Children’s Hospital, Columbus, OH USA; 56 University of Miami, Coral Gables, FL USA; 57 University of Mississippi Medical Center, Jackson, MS USA; 58 University of California, Los Angeles, Los Angeles, CA USA; 59 Medical University of Southern Carolina (MUSC), Portland, OR USA; 60 Cincinnati Children’s Hospital Medical Center-Research Foundation, Cincinnati, OH USA; 61 Seattle Children’s Autism Center/UW, Seattle, WA USA; 62 University of North Carolina at Chapel Hill, Chapel Hill, NC USA; 63 Department of Child & Adolescent Psychiatry, Rush University Medical Center, Chicago, IL USA; 64 Department of Developmental & Behavioral Pediatrics, Rush University Medical Center, Chicago, IL USA; 65 Department of Neurological Sciences, Department of Pediatrics, Department of Biochemistry, Rush University Medical Center, Chicago, IL USA; 66 Cincinnati Children’s Hospital Medical Center - Research Foundation, Cincinnati, OH USA; 67 Boston Children’s Hospital (BCH), Boston, MA USA; 68 University of North Carolina at Chapel Hill, Chapel Hill, NC USA; 69 University of Washington School of Medicine, Seattle, WA USA; 70 University of California, San Diego, School of Medicine, La Jolla, CA USA; 71 Children’s Hospital of Philadelphia, Philadelphia, PA USA; 72 Boston Children’s Hospital (BCH), Boston, MA USA; 73 University of Minnesota, Minneapolis, MN USA; 74 Kennedy Krieger Institute, Baltimore, MD USA; 75 Oregon Health & Science University, Portland, OR USA; 76 University of Minnesota, Minneapolis, MN USA; 77 University of Colorado School of Medicine, Aurora, CO USA; 78 Department of Health Psychology, University of Missouri, Columbia, SC USA; 79 Thompson Center for Autism and Neurodevelopmental Disorders, University of Missouri, Columbia, SC USA; 80 Geisinger Autism & Developmental Medicine Institute, Lewisburg, PA USA; 81 Southwest Autism Research and Resource Center, Phoenix, AZ USA

**Keywords:** Mutation, Neurodevelopmental disorders, Autism spectrum disorders, Next-generation sequencing

## Abstract

Most genes associated with neurodevelopmental disorders (NDDs) were identified with an excess of de novo mutations (DNMs) but the significance in case–control mutation burden analysis is unestablished. Here, we sequence 63 genes in 16,294 NDD cases and an additional 62 genes in 6,211 NDD cases. By combining these with published data, we assess a total of 125 genes in over 16,000 NDD cases and compare the mutation burden to nonpsychiatric controls from ExAC. We identify 48 genes (25 newly reported) showing significant burden of ultra-rare (MAF < 0.01%) gene-disruptive mutations (FDR 5%), six of which reach family-wise error rate (FWER) significance (*p* < 1.25E−06). Among these 125 targeted genes, we also reevaluate DNM excess in 17,426 NDD trios with 6,499 new autism trios. We identify 90 genes enriched for DNMs (FDR 5%; e.g., *GABRG2* and *UIMC1*); of which, 61 reach FWER significance (*p* < 3.64E−07; e.g., *CASZ1*). In addition to doubling the number of patients for many NDD risk genes, we present phenotype–genotype correlations for seven risk genes (*CTCF*, *HNRNPU*, *KCNQ3*, *ZBTB18*, *TCF12*, *SPEN*, and *LEO1*) based on this large-scale targeted sequencing effort.

## Abstract (web summary)

For many neurodevelopmental disorder (NDD) risk genes, the significance for mutational burden is unestablished. Here, the authors sequence 125 candidate NDD genes in over 16,000 NDD cases; case-control mutational burden analysis identifies 48 genes with a significant burden of severe ultra-rare mutations.

## Introduction

Neurodevelopmental disorders (NDDs) are a group of disorders primarily associated with neurodevelopmental dysfunction that include autism spectrum disorder (ASD), developmental delay (DD), intellectual disability (ID), and attention-deficit/hyperactivity disorder (ADHD)^1^. Children with NDDs experience difficulties with motor skills, learning and/or memory, language and/or nonverbal communication, and/or other neuropsychiatric problems. Considerable heterogeneity is common at both the phenotypic and genetic levels. With the advent of next-generation sequencing technologies, such as targeted sequencing^2–5^, exome sequencing^6–9^, genome sequencing^10–12^, and copy number variation (CNV) studies^13,14^, hundreds of genes and genomic regions have been implicated in NDDs almost exclusively based on the enrichment of de novo mutations (DNMs). But relatively few genes or loci have enough cases identified to prove statistical significance at the genome-wide level.

Ultra-rare and de novo gene-disruptive variants have been shown to play important roles in NDDs^15^. While DNMs from over 10,000 NDD families have been identified and cataloged^16^, the number of sequenced samples is still insufficient to reach the most stringent genome-wide significance levels, and samples from different ancestries and regions around the world are required to capture the whole picture of the genetics. Sample sizes in excess of 20,000 are projected to be necessary to reach significance levels by standard case-control criteria^17^. The discovery of large numbers of families with a disruptive variant in a specific gene, nevertheless, has facilitated establishing more meaningful genotype–phenotype correlations, such as in *CHD8*^18^, *POGZ*^19^, and *ADNP*^20^. However, relatively few ASD or NDD genes have been interrogated at this level, emphasizing the need for conducting more candidate gene studies where patients and their families can be reassessed^21^.

Using single-molecule molecular inversion probes (smMIPs) is a relatively cheap and efficient approach to target sequence candidate genes in a large number of individuals where exome or genome sequencing is not feasible, or in situations where the amount of DNA is limited^2^. Here, we present targeted sequencing using smMIPs and analysis of the coding and splicing regions of 125 NDD candidate genes in a cohort with over 16,000 NDD patients from the international Autism Spectrum/Intellectual Disability (ASID) network, which includes 18 clinical groups across the world^3^. We identify 48 genes (25 newly reported) showing significant mutation burden of ultra-rare (MAF < 0.01%) gene-disruptive mutations (FDR 5%) by comparing to ExAC nonpsychiatric controls. Among these 125 targeted genes, we also identify 90 genes enriched for DNMs (FDR 5%) by reevaluating DNM excess in 17,426 NDD trios, including 6499 new autism trios. With this large-scale targeted sequencing effort, we further double the number of patients for many NDD risk genes and present deep phenotype–genotype correlations for seven NDD risk genes (*CTCF*, *HNRNPU*, *KCNQ3*, *ZBTB18*, *TCF12*, *SPEN*, and *LEO1*).

## Results

### Targeted sequencing and variant discovery

**Fig. 1:**
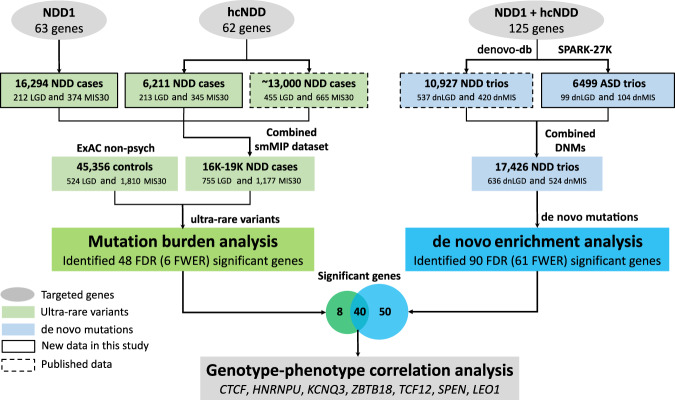
Overview of study design. Targeted sequencing was performed in probands for two gene panels: NDD1 (63 genes) and hcNDD (62 genes). Gene and variant counts are after QC. The same categories of variants were retrieved from three previously published smMIP studies for 62 hcNDD genes. All smMIP variants were combined; redundant samples were eliminated and compared to the same category of variants from ExAC non-psych controls. The number of variants is after the exclusion of false positive variants and variants with insufficient coverage in ExAC. Mutation burden analysis identified 48 FDR significant genes (*q*_mutBurden_ < 0.05, Benjamini–Hochberg correction for 125 genes), of which six reached FWER significance (*p*_mutBurden_ < 1.25E−06, Bonferroni correction for 20,000 genes and two tests); DNMs of the 125 genes used in this study were identified from exome sequencing in 10,927 published NDD trios and 6,499 new ASD trios that combined as 17,426 NDD parent–child trios. A separate de novo enrichment analysis, using two statistical methods (CH model and denovolyzeR), identified 90 FDR significant genes (*q*_dnEnrich_ < 0.05, Benjamini–Hochberg correction for 18,946 genes in CH model and 19,618 genes in denovolyzeR), of which, 61 genes reach FWER significance (*p*_dnEnrich_ < 3.64E−07, Bonferroni correction for 19,618 genes and seven tests) for excess DNM. There is a significant overlap (40 genes) of the significant genes suggested by the two approaches. Then we performed genotype–phenotype correlation analysis for seven NDD risk genes (*CTCF*, *HNRNPU*, *KCNQ3*, *ZBTB18*, *TCF12*, *SPEN*, and *LEO1*) and present a clearer clinical picture of each gene.

We initially selected 127 genes for targeted sequencing based primarily on published cases of recurrent DNM^16^, dividing the genes into two targeted sequencing panels (Fig. 1, Supplementary Data 1). The first panel (NDD1) consisted of 65 candidate genes selected for the first time in our study for sequencing in 17,832 NDD cases; the second panel (hcNDD) represented 62 genes, generally regarded as higher confidence NDD risk genes that had already been sequenced in a smaller subset (12,000–14,000) of ASID samples^3–5^. We applied this second panel to an additional 6,666 NDD cases in this study. We selected patient samples from the international ASID network of 18 clinical groups where ASD and DD/ID samples existed but neither exome nor genome sequence had been generated (Supplementary Fig. 1, Supplementary Table 1).

In panel NDD1, we designed 2,400 smMIPs to sequence the coding and splicing regions (exons plus five bases at each end) for 65 NDD candidate genes (Supplementary Data 2) among 17,832 NDD cases (8,738 and 9,094 cases with the primary diagnosis of ASD and DD/ID, respectively) (Supplementary Table 1). There were 1,538 samples (784 ASD and 754 DD/ID) and two genes (*KCNQ2* and *PAXX*) that failed quality control (QC) based on read-depth coverage statistics (Supplementary Figs. 2, 3); these samples and genes were removed from subsequent downstream analyses. In total, we identified 31,659 putative single-nucleotide variants (SNVs) or insertions/deletions (indels) for 63 genes in 16,294 samples after QC. This included 586 ultra-rare (minor allele frequency [MAF] < 0.01%, i.e., allele count [AC] ≤ 3 in this study) severe variants, where 212 were likely gene-disruptive (LGD) variants (either a frameshift, nonsense, or canonical splice donor/acceptor variant) in 241 patients, and 374 were missense variants with a Combined Annotation Dependent Depletion (CADD) score^22^ greater than or equal to 30 (MIS30) in 465 patients. Using Sanger sequencing, we validated 183 LGD variants in 204 patients and 196 MIS30 variants in 233 patients with an overall validation rate of 96.7% (379/392) (Supplementary Data 3). Transmission was successfully assessed for 110 variants where we identified 40 DNMs with 29 de novo LGD (dnLGD), 11 de novo MIS30 (dnMIS30) variants, and 70 inherited variants in 73 families (three inherited MIS30 variants observed in two unrelated families) with maternally inherited variants in 37 families (30 MIS30 and 7 LGD) and paternally inherited variants in 36 families (23 MIS30 and 13 LGD). The majority (50/70) of the inherited variants were missense mutations. Limited clinical data are available for 28 carrier parents (Supplementary Data 5). Among the families where the parental phenotype data is available, one proband also carries a de novo missense variant (p.Arg1241Gln, CADDv1.3 = 15.4) in *SHANK2* in addition to the paternally transmitted stop-gain variant (p.Arg860Ter) in *CDK13*, although the de novo variant is more likely to contribute to the proband’s autism. Most of the carrier parents (24/28) were classified as unaffected with no cognitive impairment, autism, or other psychiatric problems. The remaining four carrier parents show some clinical features related to the variant. One father, for example, who transmitted a MIS30 variant (p.Ser242Phe) in *HNRNPR*, had special education needs as he attended a school for individuals with learning disabilities but showed no obvious dysmorphic features. Similarly, a mother who transmitted a MIS30 variant (p.Arg339Gln) in *CTCF* showed a similar facial phenotype as the child but did not present with a clinical diagnosis of ID or ASD and was known to have attended regular school. A mother who transmitted a severe missense variant (p.Arg330Leu) in *KCNQ3* was diagnosed with epilepsy but no cognitive impairment (Supplementary Data 5). Finally, one mother who transmitted a splice acceptor variant (c.1189-2 A > G) in *TCF12* was diagnosed with long QT syndrome and glaucoma (like the patient) but this shared feature is unlikely related to DD observed in the child or the variant in question. These findings are consistent with the idea that such transmitted variants are by themselves not necessary and sufficient to develop DD but may rather be predisposing variants with a subset of parents manifesting more subtle phenotypes^23^.

In panel hcNDD, we resequenced 62 genes selected from our previous smMIP panels (Supplementary Data 1) for targeted sequencing with 3,575 smMIPs in 6,666 newly recruited NDD cases (3,562 ASD and 3,104 DD/ID) (Supplementary Table 1). All genes passed QC, but 455 DNA samples (199 ASD and 256 DD/ID) failed QC based on sequence coverage and were excluded from downstream analyses (Supplementary Figs. 2, 3). In total, we identified 72,811 SNV/indel variants for 62 genes in 6,211 patients after QC, including 213 LGD variants in 242 patients and 345 MIS30 variants in 426 patients. We validated 161 LGD variants in 172 patients and 170 MIS30 variants in 196 patients with a validation rate of 98.2% (331/337) for variants where Sanger sequencing was performed (Supplementary Data 3). Inheritance was assessed for 81 variants identifying 29 DNMs (21 dnLGD and 8 dnMIS30 variants) and 52 inherited (34 maternal and 18 paternal) variants. Ultra-rare severe variants were enriched ~2.5-fold among the hcNDD genes when compared to NDD1 genes for LGD (*p* = 4.82E−24, OR = 2.56 [2.14–3.08, 95% CI]) and MIS30 (*p* = 8.35E−39, OR = 2.49 [2.17–2.86, 95% CI]) variants (two-sided Fisher’s exact test), which reconfirms that these high-confidence genes usually have more severe variants in NDD cases.

### Genes with an excess burden of ultra-rare severe variants

**Fig. 2:**
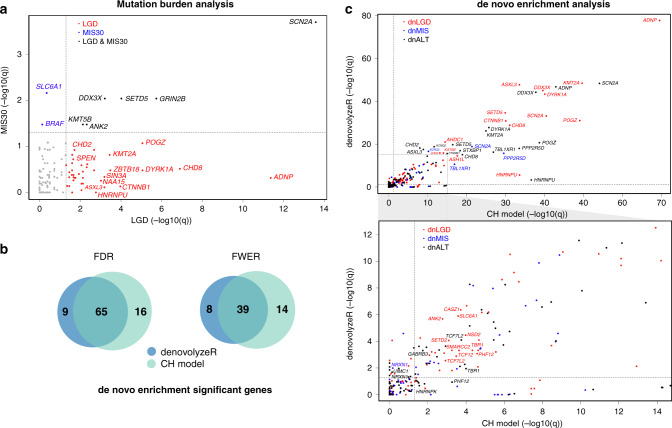
Significant genes identified from mutation burden and de novo enrichment analyses. **a** Mutation burden analysis identified 48 genes significant for LGD and/or MIS30 variants in smMIP sequencing compared with the ExAC (r0.3) non-psych subset controls; each dot indicates a gene and the color indicates the category of variant showing significance for the gene (red for LGD, blue for MIS30, and black for both LGD and MIS30). **b** The CH model and denovolyzeR show high concordance for genes with significant excess of DNM at both FDR and FWER levels. **c** A union set of 90 genes showing excess DNM (FDR 5%) in de novo enrichment analysis. Gray dashed box in top panel is shown in bottom panel for a zoom view. See Supplementary Data 10 for underlying data.

Since the 62 hcNDD genes were also previously sequenced in a subset (12,000–14,000) of ASID cases^3–5^, where we retrieved the same category of 1,120 ultra-rare severe variants with an overall similar validation rate of 97% (519/535) (Supplementary Data 4). We combined all of the retrieved data with our current sequencing in this study. Surveying the 125 genes across 16,000–19,000 NDD cases, there was a total of 2,113 ultra-rare severe variants (843 LGD and 1,270 MIS30 variants) from 2,621 patients (cases, Supplementary Data 5). In order to assess mutation burden, we extracted the same category of mutations corresponding to the smMIP capture regions for the 125 genes from ExAC (r0.3) controls^24^ without psychiatric disorders (*n* = 45,376) (controls, Supplementary Data 6). To quantify the population structure captured by our smMIPs, we conducted a principal component analysis (PCA) using the ultra-rare variants identified from our targeted sequencing, and also all the available single-nucleotide polymorphisms (SNPs) that overlap with our smMIPs from the 1000 Genomes Project (phase III high coverage) samples. We did not observe population-specific PCA clusters, suggesting that our ultra-rare variants are not stratified by different world populations (Methods). We excluded false positive variants and controlled for platform differences by removing variants with insufficient coverage between smMIP cases and ExAC controls (Methods). In total, 755 LGD and 1,177 MIS30 variants from smMIP cases, and 524 LGD and 1,810 MIS30 variants from ExAC controls were applied in the mutation burden analysis. We identified 48 genes with a significant excess of LGD and/or MIS30 (*q*_mutBurden_ < 0.05, corrected *n*_genes_ = 125, variant count > 1) (Table 1, Fig. 2, Supplementary Data 10) in cases. Of these, six genes (*ADNP*, *CHD8*, *DYRK1A*, *GRIN2B*, *POGZ*, and *SCN2A*) also reached a more stringent significance threshold that pass exome-wide Bonferroni correction at the family wise error rate (FWER) for LGD variants (*p*_mutBurden_ < 1.25E−06, corrected *n*_genes_ = 20,000, variant count > 1). Among the 48 significant genes, we identified 25 genes that show evidence of ultra-rare LGD and/or MIS30 (FDR 5%) burden for the first time in this large-scale case-control study, although 21 of these have been shown previously to show enrichment for DNMs (Supplementary Data 10).

**Table 1:** Genes with a significant burden for ultra-rare severe variants.

Gene	Samples	smMIP (AC ≤ 3) Combined (This study | Published)		ExAC non-psych (AC ≤ 9)		Mutation burden test			
		LGD	MIS30	LGD	MIS30	LGD *p*-value	MIS30 *p*-value	FDR Significance	FWER Significance
*SCN2A*	19,847	33 (12|23)	25 (4|23)	1	11	2.09E−16	1.63E−06	LGD MIS30	LGD
*GRIN2B*	19,847	14 (8|9)	14 (5|10)	0	6	5.82E−08	3.08E−04	LGD MIS30	LGD
*ADNP*	19,847	28 (13|18)	3 (1|2)	1	3	6.89E−14	2.64E−01	LGD	LGD
*CHD8*	19,847	25 (8|17)	21 (10|11)	5	32	3.03E−09	9.77E−02	LGD	LGD
*POGZ*	19,847	16 (7|9)	13 (3|11)	2	10	4.20E−07	8.24E−03	LGD	LGD
*DYRK1A*	19,847	16 (4|12)	8 (3|5)	2	9	4.20E−07	1.12E−01	LGD	LGD
*SETD5*	19,847	15 (3|12)	19 (8|15)	3	12	5.28E−06	3.68E−04	LGD MIS30	
*DDX3X*	19,847	10 (4|7)	7 (4|3)	1	0	5.41E−05	2.41E−04	LGD MIS30	
*ANK2*	19,538	17 (4|14)	61 (29|46)	11	86	7.61E−04	2.16E−03	LGD MIS30	
*KMT5B*	19,538	7 (1|6)	8 (3|7)	1	2	1.32E−03	1.63E−03	LGD MIS30	
*CTNNB1*	19,847	10 (1|9)	5 (0|5)	0	11	6.80E−06	5.65E−01	LGD	
ZBTB18*	16,321	8 (8|-)	3 (3|-)	0	2	2.37E−05	1.19E−01	LGD	
*KMT2A*	19,077	13 (5|9)	29 (9|22)	3	42	2.86E−05	2.82E−02	LGD	
ASXL3*	19,077	11 (1|10)	3 (0|3)	2	7	6.32E−05	6.06E−01	LGD	
*SIN3A*	19,538	8 (2|6)	12 (3|10)	0	20	6.73E−05	2.32E−01	LGD	
*NAA15*	19,538	13 (1|12)	6 (3|3)	4	10	1.07E−04	3.43E−01	LGD	
HNRNPU*	16,321	8 (8|-)	0 (0|-)	2	0	6.26E−04	1	LGD	
*DSCAM*	19,847	11 (4|8)	43 (21|34)	3	64	2.83E−04	2.01E−02	LGD	
*TRIO*	19,847	11 (6|5)	22 (8|15)	3	35	2.83E−04	1.17E−01	LGD	
WAC*	19,847	9 (2|7)	12 (1|12)	2	14	6.49E−04	6.65E−02	LGD	
RELN*	19,847	11 (3|8)	45 (23|37)	4	78	7.68E−04	8.43E−02	LGD	
PASK*	19,077	41 (17|29)	9 (5|6)	50	12	1.28E−03	1.38E−01	LGD	
ZMYM2*	19,077	11 (7|7)	4 (2|2)	5	5	1.38E−03	2.61E−01	LGD	
*SMARCC2*	19,847	7 (0|7)	4 (0|4)	1	7	1.42E−03	4.43E−01	LGD	
KAT6A*	16,321	8 (8|-)	7 (7|-)	3	24	1.76E−03	7.49E−01	LGD	
CHAMP1*	16,321	6 (6|-)	1 (1|-)	1	1	1.84E−03	4.59E−01	LGD	
*ASH1L*	19,847	10 (5|6)	39 (17|28)	4	65	1.88E−03	7.38E−02	LGD	
*NFIA*	19,847	5 (2|4)	3 (1|2)	0	2	2.61E−03	1.69E−01	LGD	
MYT1L*	19,077	7 (0|7)	10 (4|6)	2	17	3.94E−03	2.57E−01	LGD	
*DLG4*	19,538	7 (2|5)	7 (2|5)	2	11	4.38E−03	2.81E−01	LGD	
CHD2*	19,847	8 (5|4)	17 (3|16)	3	18	4.73E−03	1.84E−02	LGD	
NEXMIF*	16,321	6 (6|-)	3 (3|-)	2	3	5.71E−03	1.93E−01	LGD	
BRPF1*	16,321	5 (5|-)	13 (13|-)	2	27	1.65E−02	2.40E−01	LGD	
PHF12*	16,321	6 (6|-)	8 (8|-)	2	17	5.71E−03	3.32E−01	LGD	
SATB2*	16,321	5 (5|-)	4 (4|-)	1	7	6.02E−03	3.26E−01	LGD	
SPEN*	16,321	9 (9|-)	31 (31|-)	6	57	6.25E−03	4.26E−02	LGD	
AHNAK*	19,538	26 (11|17)	11 (5|9)	30	10	7.26E−03	2.70E−02	LGD	
ZNF292*	19,077	9 (3|6)	0 (0|0)	5	6	7.52E−03	1	LGD	
PHIP*	19,847	10 (3|7)	18 (3|15)	6	24	7.96E−03	5.96E−02	LGD	
*TNRC6B*	19,847	10 (2|8)	12 (2|10)	6	16	7.96E−03	1.12E−01	LGD	
KMT2E*	19,538	9 (5|5)	10 (3|7)	5	15	8.48E−03	1.92E−01	LGD	
*TRIP12*	19,847	7 (1|6)	16 (8|10)	3	25	1.15E−02	1.52E−01	LGD	
TBR1*	19,847	5 (2|3)	3 (2|1)	1	6	1.17E−02	5.49E−01	LGD	
SETBP1*	19,847	6 (2|4)	9 (7|5)	2	14	1.22E−02	2.43E−01	LGD	
PHF7*	19,538	9 (4|5)	3 (3|0)	6	6	1.56E−02	5.40E−01	LGD	
TCF12*	16,321	9 (9|-)	12 (12|-)	8	18	1.79E−02	7.34E−02	LGD	
*SLC6A1*	19,847	1 (0|1)	18 (8|12)	0	9	3.04E−01	1.11E−04	MIS30	
BRAF*	16,321	1 (1|-)	11 (11|-)	2	7	6.02E−01	2.03E−03	MIS30	

Fisher’s exact test (one-sided) for LGD and MIS30 variants from smMIP sequencing compared to the ExAC (r0.3) non-psych subset identified 48 genes significant at the FDR level, of which, six genes reach FWER significance. The FDR significance threshold *q*_mutBurden_ < 0.05 was corrected by the Benjamini–Hochberg method for 125 genes in this study; the FWER significance threshold *p*_mutBurden_ < 1.25E−06 was corrected by the Bonferroni method for 20,000 genes in human genome and two tests performed (LGD and MIS30 variants). *Indicates 25 genes showing new mutational burden significance in case-control analysis of ultra-rare LGD and MIS30 variants in this study. See Supplementary Data 10 for underlying data.

### Reevaluation of genes for excess DNMs

As the parent–child exome sequencing for ASD and DD/ID families has increased since the original selection of candidate genes, we also reassessed each of the 125 genes for excess DNM in a larger NDD combined set. In addition to the 537 dnLGD variants and 420 de novo missense (dnMIS) variants from previously published 10,927 NDD cases^25^ (Supplementary Data 8), we identified 99 dnLGD and 104 dnMIS (including 31 dnMIS30) variants in 6,499 new ASD patients from 5,911 complete families (4,761 simplex and 1,150 multiplex families) in our recent analysis of 27,270 SPARK exomes (unpublished data, https://sparkforautism.org/) (Supplementary Data 9). In total, there are 636 dnLGD and 524 dnMIS (including 201 dnMIS30) variants in the 125 genes from 17,426 NDD (12,123 ASD and 5,303 DD/ID) cases. We reevaluated the genes for excess DNM (dnLGD, dnMIS, dnMIS30, or de novo protein alteration [dnALT] variants that include dnLGD and dnMIS) using two statistical models (Fig. 1): a modified chimpanzee–human divergence model (CH model)^4^ and the denovolyzeR^26^ model as previously described^25^. Correcting for the total number of genes in each model, 81 genes show excess DNM in NDD patients according to the CH model (*q*_dnEnrich_ < 0.05, corrected *n*_genes_ = 18,946, DNM count > 1) compared to 74 genes predicted to be enriched by denovolyzeR (*q*_dnEnrich_ < 0.05, corrected *n*_genes_ = 19,618, DNM count > 1) (Fig. 2, Supplementary Data 10). The combination of both models identified 90 significant NDD candidate genes (union), and 65 genes were seen by both models (intersection). Applying a more stringent FWER significance (*p*_dnEnrich_ < 3.46E−07, corrected *n*_genes_ = 19,618 in seven tests, DNM count > 1) identifies 61 union genes and 39 intersect genes (Fig. 2, Supplementary Data 10). This includes two genes (*UIMC1* and *GABRG2*) firstly significant at a 5% FDR and seven genes (*ANK2*, *TBR1*, *PHF12*, *TCF7L2*, *SETD2*, *CASZ1*, and *NSD2*), which were significant at 5% FDR previously, that firstly reach FWER significance in this larger NDD cohort (Table 2, Supplementary Data 10).

**Table 2:** Genes reaching new de novo enrichment significance.

Gene	DNM All (denovo-db | SPARK-27K)			CH model				denovolyzeR			Significance (union of two models)		Reported significance		
	dnLGD	dnMIS	dnMIS30	dnLGD *p*-value	dnMIS *p*-value	dnMIS30 *p*-value	dnALT *p*-value	dnLGD *p*-value	dnMIS *p*-value	dnALT *p*-value	FDR	FWER	Coe253	ASC102	DDD299
*New FDR significance*															
*UIMC1*	0 (0|0)	6 (4|2)	0 (0|0)	1	1.67E−02	1	2.94E−02	1	5.00E−05	2.01E−04	dnMIS dnALT	–	No	No	No
*SMARCC2*	4 (2|2)	2 (2|0)	0 (0|0)	6.53E−05	4.44E−01	1	8.20E−03	5.17E−05	3.76E−01	4.49E−03	dnLGD	–	No	Yes	No
*NRXN1*	1 (1|0)	7 (5|2)	1 (1|0)	2.13E−01	4.70E−03	2.06E−01	2.30E−03	1.57E−01	1.54E−03	4.87E−04	dnALT	–	No	Yes	No
*HNRNPK*	2 (2|0)	2 (1|1)	2 (1|1)	1.16E−03	3.89E−02	2.30E−04	5.00E−04	6.27E−03	9.96E−02	4.38E−03	dnALT	–	No	No	Yes
*GABRG2*	1 (0|1)	4 (3|1)	3 (3|0)	2.41E−01	1.52E−01	1.29E−03	8.69E−02	1.22E−01	1.47E−03	4.16E−04	dnALT	–	No	No	No
*New FWER significance*															
*ANK2*	9 (6|3)	5 (5|0)	1 (1|0)	1.13E−05	9.97E−01	6.88E−01	6.08E−01	6.09E−09	3.03E−01	1.22E−04	dnLGD dnALT	dnLGD	Yes	Yes	No
*TBR1*	4 (3|1)	3 (2|1)	1 (0|1)	1.93E−07	9.30E−03	2.60E−02	6.81E−07	2.01E−06	6.88E−02	9.41E−05	dnLGD dnALT	dnLGD	Yes	Yes	No
*PHF12*	4 (3|1)	1 (1|0)	0 (0|0)	1.06E−07	3.02E−01	1	6.08E−05	4.00E−06	6.83E−01	9.15E−03	dnLGD dnALT	dnLGD	Yes	Yes	No
*TCF7L2*	4 (3|1)	5 (3|2)	3 (3|0)	7.36E−06	2.30E−03	3.70E−04	1.26E−06	1.56E−05	9.05E−04	3.47E−07	dnLGD dnALT	dnALT	Yes	Yes	Yes
*SETD2*	6 (5|1)	2 (2|0)	1 (1|0)	4.39E−06	8.43E−01	2.56E−01	3.59E−02	3.30E−07	6.63E−01	4.55E−03	dnLGD	dnLGD	Yes	No	Yes
*CASZ1*	6 (4|2)	2 (0|2)	1 (0|1)	8.43E−07	8.17E−01	2.17E−01	2.35E−02	1.16E−09	7.09E−01	5.10E−03	dnLGD	dnLGD	Yes	No	No
*NSD2*	5 (4|1)	1 (1|0)	1 (1|0)	4.57E−07	7.20E−01	1.35E−01	3.40E−03	1.19E−07	7.82E−01	6.60E−03	dnLGD	dnLGD	Yes	No	Yes

Five genes newly reached FDR significance and seven genes reached FWER significance in the de novo enrichment analysis, compared to Coe et al.^25^, using the same methods (CH model and denovolyzeR) with DNMs in 17,426 NDD trios combined from denovo-db (v1.5) and SPARK-27K. The FDR significance threshold *q*_dnEnrich_ < 0.05 was corrected by the Benjamini–Hochberg method for genes in each method (18,946 genes in CH model and 19,618 genes in denovolyzeR); the FWER significance threshold *p*_dnEnrich_ < 3.64E−07 was corrected by the Bonferroni method for 19,618 genes and seven tests (dnLGD, dnMIS, dnMIS30, and dnALT variants in CH model, and dnLGD, dnMIS, and dnALT variants in denovolyzeR). Coe253 indicates whether the gene is in the 253 genes reported significant (FDR 5%) in Coe et al.^25^; ASC102 indicates whether the gene is in the 102 genes reported as significant (FDR 10%) in Satterstrom et al.^8^; and DDD299 indicates whether the gene is in the 299 genes reported as significant in Kaplanis et al.^31^. Note different methods and significant threshold were applied in those three studies. See Supplementary Data 10 for underlying data.

### Genotype–phenotype correlations

**Fig. 3:**
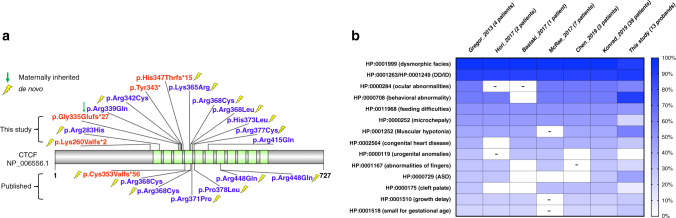
Severe variants and the genotype–phenotype correlations in *CTCF*. **a** LGD (red) and MIS30 (blue) variants are depicted against a protein model for *CTCF*. Variants new to this study are shown above the protein while published DNMs from denovo-db (v1.5) are below. Variants are flagged with yellow lightning bolt if de novo. Annotated protein domains are shown (colored blocks) for the largest protein isoforms. **b** Heatmap depicts the common clinical features for patients carrying *CTCF* severe variants by using the specific HPO annotation (rows), which were retrieved from published studies and our cohort (columns). Phenotypic enrichment is shown according to the features’ recurrence labeled by the increment of color degree. The items with no data available were labeled with “-” and were excluded in the frequency analysis.

**Fig. 4:**
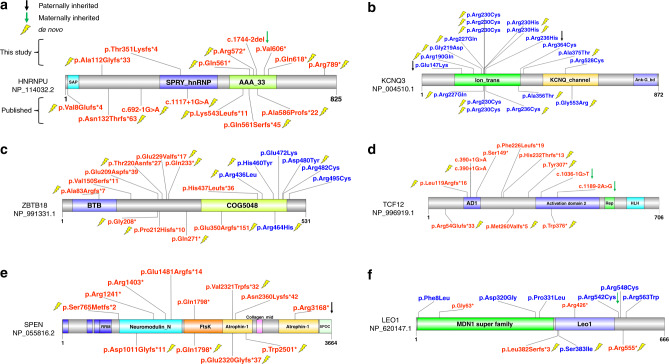
Distribution of severe patient variants in six genes. Protein diagrams are shown for *HNRNPU* (**a**) *KCNQ3* (**b**) *ZBTB18* (**c**) *TCF12* (**d**) *SPEN* (**e**), and *LEO1* (**f**) with the same display metrics that applied in Fig. 3. Validated LGD (red) and MIS30 (blue) variants are plotted. Variants listed above the protein model are new to this study, while the ones below were published previously. Paternal (black arrow) and maternal (green arrow) inheritance are shown if determined. A yellow lightning bolt denotes a de novo mutation.

We successfully collected clinical records for 41 probands that carry ultra-rare severe variants in seven significant genes (*CTCF*, *HNRNPU*, *KCNQ3*, *ZBTB18*, *TCF12*, *SPEN*, and *LEO1*) from families that were available for recontact (Figs. 3 and 4, Supplementary Data 11). We also obtained clinical information for nine probands with dnMIS variants (2 in *CTCF*, 4 in *KCNQ3*, and 3 in *ZBTB18*) identified from the clinical trio exome sequencing at Baylor Genetics, and one DD patient with a dnLGD variant in *CTCF* that was identified from trio exome sequencing by the Antwerp group (Supplementary Data 11). We integrated the above clinical records with previously published reports and present a more comprehensive genotype–phenotype correlation assessment within the context of each gene (Table 3, Supplementary Data 12–18).

**Table 3:** Clinical recontact and detailed genotype–phenotype correlations.

Gene	CTCF	HNRNPU	KCNQ3	ZBTB18	TCF12	SPEN	LEO1
OMIM gene	*604167	*602869	*602232	*608433	*600480	*613484	*610507
OMIM phenotype	#615502	#617391	#121201	#612337	#615314	NR	NR
Inheritance pattern	AD	AD	AD	AD	AD	NR	NR
# Patients	~70	~35	~46	~31	~124	~10	~8
Clinical synopsis (most frequent features)	Microcephaly, thin vermilion border, Abnormality of the dentition; hypermetropia, strabismus, delayed dentition. Feeding difficulties. Congenital cardiopathies. Cryptorchidism. Hypotonia, global developmental delay, intellectual disability. Growth delay and short stature.	Microcephaly. Generalized hypotonia. Delayed myelination, EEG abnormality, epileptic encephalopathy, global developmental delay, intellectual disability, ventriculomegaly.	Benign familial neonatal epilepsy and benign familial infantile epilepsy, seizure disorders that occur in children who typically have normal psychomotor development. Developmental disability with or without seizures and/or cortical visual impairment.	Moderate to severe intellectual disability, limited or no speech, and variable but characteristic facial features including a round face, prominent forehead, flat nasal bridge, hypertelorism, epicanthal folds, and low-set ears. Hypotonia, poor growth, microcephaly, agenesis of the corpus callosum, and seizures.	Variable craniosynostosis that may involve, individually or in combination, the coronal and/or the sagittal skull sutures. Other congenital anomalies, dysmorphisms (brachydactyly, ptosis, strabismus) and/or neurodevelopmental impairment may be present.	Mild facial dysmorphisms, muscular hypotonia, tall stature, poor motor coordination, and ocular abnormalities	Intellectual disability and autistic behavior

Data were retrieved by analyzing available clinical reports for genes of interest, and a clinical synopsis is presented according to MedGen. Individual patient details can be found in Supplementary Data 12–18, respectively.

*AD* autosomal dominant, *NR* not reported.

Germline deleterious variants in *CTCF* have recently been implicated in autosomal dominant DD/ID syndromic disorder (OMIM #615502) (Supplementary Data 12) with clustering of dnMIS30 variants occurring near the zinc-finger DNA binding domains associated with this protein^27^. We assessed 13 additional probands (including six with clustered dnMIS variants) from our study (Fig. 3). They are characterized by craniofacial dysmorphisms (9/10), thin vermillion border and lips (4/7), and feeding difficulties (6/11), and exhibit neonatal hypotonia (10/7). Along with these features, patients with *CTCF* mutations display a broader spectrum of developmental anomalies, including cardiac congenital malformations (1/8) and skeletal anomalies of toes/fingers (2/10). In addition to DD/ID (11/12), 54.5% (6/11) of the patients have a diagnosis of ASD and/or ADHD. The incidence of each phenotype in our probands (*n* = 13) is representative of the combined dataset, including published reports (*n* = 56) (Fig. 3).

*HNRNPU* mutations are now recognized as causative for early infantile epileptic encephalopathy-54 (EIEE54) syndrome (OMIM #617391), also referred to as *HNRNPU*-related disorder^28^. We observed seizures (3/3), DD/ID and ASD comorbidities (3/3), movement disorders such as stereotypies, e.g., hand flapping (1/3), and severe speech impairment (1/3) among our patients (Supplementary Data 13). We observed high ASD comorbidity (5/9) in patients carrying *KCNQ3* mutations extending the phenotype which primarily associated with benign familial neonatal epilepsy. In our study, about half of the patients were diagnosed with benign familial infantile epilepsy (4/9) or DD (5/9) with or without seizures and cortical visual impairment (Supplementary Data 14). In contrast to *HNRNPU*, all mutations associated with *KCNQ3* were severe missense mutations with no observation of a potential LGD mutation^29^. *ZBTB18* is responsible for autosomal dominant mental retardation-22 (MRD22) syndrome (OMIM #612337), which is characterized by the features also seen in our patients such as moderate to severe DD/ID (7/7), ASD (2/7), speech delay (2/4), variable facial dysmorphisms (3/3), growth delay (2/4), and poor fine-motor skills (2/4) (Supplementary Data 15). *TCF12* has been associated with craniosynostosis-3 syndrome (OMIM #615314). This phenotypic feature was observed in two of our patients, as well as other neurobehavioral phenotypes (DD/ID in 3/8 and ASD in 4/8 patients) (Supplementary Data 16).

We also investigated two additional candidate genes: *SPEN* and *LEO1*. To our knowledge, *SPEN* is newly identified in this study with a significant burden only for LGD variants (Table 1), while *LEO1* shows excess DNM at both FDR and FWER levels (Supplementary Data 10). All patients with deleterious variants in *SPEN* show neurobehavioral impairment (Supplementary Data 17) (e.g., DD/ID in 6/7 and ASD in 5/7 patients in this study). Patients with a deleterious variant in *SPEN* show a more complicated clinical picture with other features, such as mild facial dysmorphism (4/4), muscular hypotonia, tall stature, poor motor coordination, and ocular abnormalities (3/4). Paternally inherited deletions of the *LEO1* promoter were recently detected in three individuals with ASD^11^. Only two patients with disruptive mutations in *LEO1* from our cohort could be recontacted, one showed some dysmorphic features and a minor cardiopathy plus global DD, while the other showed rather non-syndromic neurobehavioral features (Supplementary Data 18).

## Discussion

Here, we report the results of large-scale targeted sequencing of 125 genes in over 16,000 pediatric NDD patients, with more than half the genes being screened in over 19,000 patients. We investigate these genes under a case-control mutation burden design and also test for DNM enrichment. Our comparison to ExAC controls identifies 48 genes as significantly enriched for ultra-rare severe variants in NDD patients (LGD and/or MIS30 variants, *q*_mutBurden_ < 0.05, corrected *n*_genes_ = 125, variant count > 1). Additionally, 90 of the genes are enriched for DNMs in combined exomes of 17,426 NDD parent–child trios. There are 40 genes significant in both tests defining a subset of genes particularly relevant for future diagnosis of disease irrespective of inheritance patterns or availability of parental data. Overall, 78.4% (98/125) of the genes show some evidence of mutational burden in patients; notably, 61 genes remain significant at a more stringent level of FWER significance (61 with de novo enrichment, six of which were also detected from the case-control design) (Supplementary Data 10). In our targeted sequencing, 76% (95/125) of these genes have ultra-rare LGD variants identified in both patients with a primary diagnosis of ASD and DD/ID suggesting that these particular genes should be regarded as NDD genes as opposed to solely ASD or DD/ID risk genes.

In addition to the 98 genes significant by mutation burden analysis, or the de novo enrichment analysis, or both, there are additional candidates that trend toward increased mutational burden or de novo enrichment among NDD cases. For example, there are seven additional genes if considering a less stringent threshold (FDR 10%). One gene, *NCKAP1*, shows evidence of increased mutational burden for LGD variants (*q*_mutBurden_ = 0.07), while six genes show excess DNM, namely *SF3B1* (dnMIS *q*_dnEnrich_ = 0.068 and dnALT *q*_dnEnrich_ = 0.074), *H2AC6* (dnMIS *q*_dnEnrich_ = 0.053), and *NFIA* (dnALT *q*_dnEnrich_ = 0.086) in the CH model and *ARID2* (dnLGD *q*_dnEnrich_ = 0.094), *TNRC6B* (dnLGD *q*_dnEnrich_ = 0.097), and *DNM1* (dnLGD *q*_dnEnrich_ = 0.071) under the denovolyzeR model. Given the reported function of these genes and published case reports, it is likely that with increasing sample size these genes may achieve significance in the near future^25^. To test this, we expanded the number of parent–child trio exome sequencing cases with those from the SPARK pilot study^30^ and two recent publications from the ASC study^8^ and DDD study^31^ for a total of 48,281 NDD trios (excluding sample overlap and redundancy). Across those samples, four of the seven candidate genes reach some level of significance: *ARID2* and *DNM1* are significant for excess DNM at FWER significance, and *H2AC6* and *SF3B1* show excess DNM (FDR 5%). Overall, in this expanded de novo enrichment analysis, we estimate that at least 102 of the 125 genes in this study show a significant excess of DNM after adding the SPARK pilot, ASC, and DDD cohorts. Importantly, as additional genes become significant, our targeted sequencing studies will provide an important resource for future follow-up with clinicians and additional families to further investigate these genes.

We followed up clinically on seven candidates with the aim to develop or extend genotype–phenotype correlations. For example, CTCF, the CCCTC-binding factor, is a highly conserved zinc-finger protein that forms a multifunctional complex functioning in defining topologically associated domains, which are important for genome regulation and gene expression^32^. DNMs in *CTCF* have been described in patients with ID^27^. In this study, we identified three dnMIS30 variants based on smMIP screening (Supplementary Data 12) and characterized three additional DD patients with DNM in *CTCF* from the clinical trio exome sequencing at Baylor Genetics and the Antwerp group. Phenotypic assessments confirm features of the disorder and the importance of germline mutations in *CTCF* as causative for an autosomal dominant DD/ID syndromic disorder. The aggregate data highlight a striking clustering of deleterious missense mutations between the 2nd and 5th zinc-finger domain^27^ (Fig. 3). These functional domains have been described as the most important for making contact between the *CTCF* complex and DNA molecules and, as such, may represent useful targets for future therapeutic intervention^33^.

Other genes, such as *KCNQ3*, show a preponderance of severe missense mutations with half of the mutations mapping to the ion transport domain of the protein (Fig. 4). In our study, 5/9 of our patients with clinical information and a *KCNQ3* variant are diagnosed with ASD (Supplementary Data 14), expanding the phenotypic spectrum of this gene as well as the main features of DD/ID and benign familial neonatal epilepsy^34^. All three of our recontacted patients with *HNRNPU* variants present with seizures (Supplementary Data 13), consistent with its association with epileptic encephalopathy and DD^28^. All four of our patients with a putative *ZBTB18* (also known as *RP58* or *ZNF238*) LGD variant present with DD/ID (Supplementary Data 15); this particular KRAB C2H2 zinc-finger protein has been described as a transcriptional repressor critical during brain development and neuronal differentiation^35^. Besides the previously reported large number of patients with *TCF12* mutations^36^, we identified eight patients with a generally similar phenotype showing comorbid conditions of ASD and DD/ID in about half of the cases while craniosynostosis, which was originally primarily associated with this gene, was observed in only one-third of affected individuals (Supplementary Data 16).

Some of the newer candidates that have now reached or are nearing statistical significance for mutational burden still require much more extensive clinical follow-up and additional cases to further establish variant pathogenicity and refine the associated phenotype. Such is the case for RNA polymerase-associated protein LEO1, recently implicated in ASD^11^, although there are relatively few patients reported to date. We identified two additional individuals with stop-gain variants in *LEO1*, albeit with limited clinical information. Both of them are male—one patient presented with DD and the other with autistic behavior and ADHD with bilateral cryptorchidism (Supplementary Data 18). *LEO1* is particularly intriguing in light of the recent observation that LEO1 interacts with the *PAF1C* complex in *Drosophila* to selectively transcribe expanded GGGGCC repeats in *C9orf72*-associated frontotemporal degeneration^37^. In addition, paternally inherited deletions of the *LEO1* promoter^11^ and dnLGD variants in *LEO1* have been reported in large cohort testing of DD and ASD patients^7,9^.

*SPEN* is another interesting candidate for further investigation. Haploinsufficiency of *SPEN* is considered a candidate for the 1p36 deletion syndrome phenotype^38^ and complete knockout of the gene in mice results in postnatal growth retardation and hypoplasia of the brain, especially involving the hippocampus and cerebral cortex^39^. We identified seven individuals in our study with DD and/or ASD with variable degrees of clinical information (Supplementary Data 17). Families with probands with *SPEN* LGD variants have no family history of DD/ID, learning disabilities, or neurological disease. For two patients where clinical data are more extensive, there is an indication of potential dysmorphology and skeletal abnormalities similar to previous reports. While the data, taken together, support the pathogenicity of *SPEN* LGD mutations, they also highlight a challenge going forward for the community. Unlike genes such as *CHD8*, *POGZ*, and *ADNP*, where large-scale screening has uncovered dozens of affected individuals for clinical evaluation and proved statistical significance at every level, the next tier of genes with ultra-rare and gene-disruptive DNMs will likely require screening of over 100,000 people. If only a handful of individuals with mutations in such genes are available, either from disparate labs with different standards of clinical reporting, or with incomplete family data, the pathogenicity determination may languish for years. Since we estimate that this next tranche of genes may account for more than half of the de novo gene burden associated with NDDs^6^, a more systematic effort involving targeted resequencing of large cohorts, database coordination (e.g., GeneMatcher), and dedicated researchers/clinicians willing to adopt such orphan genes and collate the clinical data are key. To help avoid false associations, whole-genome sequencing of such patients, their families, and controls may be particularly important to eliminate other genetic causes as contributing to disease and to understand the penetrance of the mutations under study.

## Methods

### Candidate genes

We considered two sets of genes: new candidates (NDD1) for investigation and high-confidence genes (hcNDD) that have been previously implicated in NDDs. Different criteria were used in selecting these two groups. In panel NDD1, we ranked and selected candidate genes for which no smMIP sequencing had been performed previously. We initially ranked all genes based on the DNMs from published NDD trios cataloged in denovo-db (v1.5), but excluding the following: genes associated with well-known syndromes based on OMIM, genes with extremely high-GC content, and genes with high counts of LGD and MIS30 variants in the ExAC non-psych controls. In total, 65 genes were selected for screening with: (i) 43 genes showing excess DNM^25^; (ii) 14 genes with evidence of autism sex bias^40^; (iii) six genes from a network analysis of high-functioning autism indicated previously^3^; (iv) and two genes (*H2AC6* and *H1-4*) that were considered within a CNV candidate. In panel hcNDD, we continually reselected 62 top candidate genes from our previous smMIP panels^3^, mainly ranked by the reported number of DNMs from the published NDD trios in denovo-db (v1.5) and number of ultra-rare severe LGD and MIS30 variants identified in targeted sequencing of >13,000 NDD cases. We sequenced an additional 6666 newly recruited NDD cases that had not been previously sequenced using smMIPs. These served as positive controls of known disease genes in this study allowing for the discovery of additional cases for phenotypic evaluation. During the selection of these 125 genes, we evaluated the success rate of all smMIPs for each gene as part of our optimization experiments. We excluded genes, for example, where >20% of smMIPs failed to provide sufficient coverage even after 50-fold spike-in. We also balanced the total number of smMIPs per gene in each panel needed to achieve sufficient sequence depth. In particular, large genes requiring more than 200 smMIPs were triaged to allow a greater number of more moderate-sized genes to be considered. Supplementary Data 1 lists the genes with detailed selection criteria.

### Study samples

Patient samples were obtained from the ASID network with informed consent. Only those not previously exome or genome sequenced were selected for targeted sequencing in this study. ASID is an international consortium^3^ that has expanded to include 18 centers around the world (Supplementary Fig. 1, Supplementary Table 1). The majority of samples were recruited from four sites (Adelaide, ACGC, Troina, and Leuven), as well as three new recruitment centers: an ASD collection from the University of Iowa (Iowa), an ID cohort from Charles University of Czech Republic (Charles), and an ASD cohort from the Italian Autism Network (ITAN). All targeted sequencing, Sanger variant validation, transmission analysis, and clinical recontact performed on the individuals in this study were approved by the University of Washington Institutional Review Board (IRB), in accordance with the ethical standards of the responsible local institutional and national committees. A PCA was used to quantify the population structure captured by our smMIPs. Samples in NDD1 generated two clusters; however, each cluster was composed of samples with mixed ancestries, and 15,659 samples (i.e., 96.1% of the total) were located under one heterogeneous cluster. In the case of hcNDD, a total of three clusters are observed; however, one of the clusters contains a heterogeneous mixture of 6161 samples (99.2% of the total). Overall, these observations suggest that the ultra-rare variants assayed by our targeted sequencing do not capture underlying population structure. Indeed, when we used all the available SNPs that overlap with our smMIPs from the 1000 Genomes Project (phase III high coverage) samples, we observed one large PCA cluster where 2,484 (99.2%) of the samples were included, once again supporting our previous observation that the genotypes of our ultra-rare variants are not stratified by different world populations. Hence, we expect our downstream case-control mutation burden analyses of the ultra-rare variants to not be confounded by population structure.

### Targeted sequencing

All of the smMIP capture experiments, HiSeq 2500 sequencing, and Sanger validation experiments were performed at the University of Washington (Seattle, WA, USA), except for the ACGC cohort where experiments were carried out at the Center of Medical Genetics, School of Life Sciences, Central South University, Changsha, Hunan, China. In NDD1, 2,400 smMIPs were designed using MIPgen^41^ to cover all annotated RefSeq protein-coding exons and the splicing portions within 5 bp of flanking intronic sequence for all 65 genes. Oligos were ordered from Integrated DNA Technologies (IDT, https://www.idtdna.com/). smMIPs were pooled, rebalanced, and spiked-in at a relative concentration of 10X or 50X to improve sequence coverage for poorer-performing smMIPs where possible (Supplementary Data 2). A total of 17,832 NDD cases were sequenced using the balanced NDD1 panel. For hcNDD, 3,575 smMIPs from 62 genes were re-pooled from previous designs^3^ and tested for 6,666 newly collected NDD cases. smMIP capture libraries were barcoded and pooled with ~288 (3 × 96) samples and sequenced on a lane using an Illumina HiSeq 2500.

### Variant annotation and validation

HiSeq data were processed according to the manufacturer’s instructions for base calling; variants were called using FreeBayes (version 1.0.2-6-g3ce827d) with its simplest operation (freebayes -f ref.fa aln.bam > var.vcf). Variants were filtered (QUAL > 20 and DP > 8) excluding common variants in dbSNP142 and then annotated using Ensembl’s Variant Effect Predictor^42^ (VEP, Ensembl GRCh37 release 94 - October 2018) with assembly GRCh37.p13 as the reference genome. Variants were annotated for all isoforms by VEP and those with the most severe consequence were selected for follow-up. Sanger validations were performed with ~300 bp PCR amplicons. CADD (v1.3) is a tool for scoring the deleteriousness of SNVs as well as indels in the human genome, and MIS30 variants are among the top 0.1% of the ~8.6 billion SNVs of the GRCh37/hg19 reference genome. LGD and MIS30 variants for the 62 genes in hcNDD were obtained from three previously published smMIP studies with same criteria applied (QUAL > 20, DP > 8, and MAF < 0.01% (AC ≤ 3)) (Supplementary Data 4). Similar variants from the targeted regions of 125 genes were obtained from the ExAC non-psych subset as controls with same filtering, i.e., QUAL > 20, DP > 363,008 (Avg. DP > 8), and MAF < 0.01% (AC ≤ 9) (Supplementary Data 6). All smMIP variants (this study and published) were merged with redundancy removed as variants with AC ≤ 3 retained for all subsequent analyses. dnLGD and dnMIS variants in the de novo enrichment analysis were extracted from SPARK-27K cases with ASD (*n* = 6,499) from complete families and the denovo-db (v1.5) NDD subset (*n* = 10,927). The published exome DNMs from SPARK pilot and ASC, together with recently released exome DNMs from DDD, were also included in the extended de novo enrichment analysis with sample overlap and redundancy removed. For cohorts like SSC and SPARK, for which the underlying exome data are available, duplicates were identified by running the KING software^43^, which uses identical by state (IBS) to estimate pairwise relatedness between samples. Any samples with a kinship value > 0.35 were considered to be identical and counted only once. Identical samples from the same cohort were also checked for reported monozygotic twin status. We identified one pair of SSC samples and eight pairs of SPARK samples as having a kinship value > 0.35. Note, samples in SPARK that overlapped with SSC samples were already removed in the final release by the SPARK Consortium. For other published cohorts, for which the underlying exome data are unavailable, the potential sample overlap identification, if applied, was described in each corresponding study. Like in the current DDD study, a total of eight duplicate samples were identified by collecting genotypes at 47 common exonic SNPs for every sample with a DNM found in another individual in the joint set; only one individual from each duplicate pair was kept with a final set of 31,058 samples analyzed. We also excluded sample overlaps reported in the literature. We excluded DD/ID samples in denovo-db (v1.5), which are also included as part of the current DDD study, and also excluded all 2,384 SSC samples in the ASC paper for potential redundancy with denovo-db (v1.5). These measures yielded a total of 48,281 NDD trios in the extended de novo enrichment analysis. To ensure uniformity, the same version of CADD score and VEP annotation were applied, and the analysis was restricted to the canonical transcript with the most deleterious annotation.

### Statistical analyses

All statistical tests were performed using the R programming language (version 3.6.1). Benjamini–Hochberg FDR or Bonferroni FWER was applied when appropriate for multiple testing correction as described in the relevant sections. For mutation burden analysis, Fisher’s exact test (one-tailed) was used to compare the number of LGD and MIS30 variants from smMIP sequencing (cases) with those from the ExAC non-psych subset (controls), false positive variants by Sanger validation and variants with insufficient coverage (<90% samples with at least 10X coverage) in ExAC were excluded. The FDR significance threshold was set as *q*_mutBurden_ < 0.05 where the *q*-value was corrected by Benjamini–Hochberg method for the total number of genes in this study (n_genes_ = 125); the FWER significance threshold was set as *p*_mutBurden_ < 1.25E−06, which was calculated by 0.05/(20,000*2) and corrected by Bonferroni method for 20,000 genes in human genome and two tests performed (LGD and MIS30 variants). For de novo enrichment analysis, we applied both the CH model^2^ and denovolyzeR^26^ methods to assess the enrichment for four classes of DNM: dnLGD, dnMIS, dnMIS30, and dnALT. We applied denovolyzeR (v0.2.0) using default settings where dnMIS30 variants are not assessed; a modified CH model^4^ was applied to include the evaluation of dnMIS30 variants. Both methods apply their own underlying mutation rate estimates to generate the prior probabilities for observing a specific number and class of mutations for a given gene. Briefly, the CH model estimates the number of expected DNMs by incorporating chimpanzee–human coding sequence divergence and the length of the gene; denovolyzeR estimates mutation rates based on trinucleotide context, mutational biases such as CpG hotspots, and macaque–human gene comparisons. Default parameters were used for both methods, and the expected mutation rate of 1.8 DNMs per exome was set to the CH model as an upper bound baseline. The FDR significance threshold was set as *q*_dnEnrich_ < 0.05 and corrected by the Benjamini–Hochberg method for the number of genes in each model (18,946 for CH model and 19,618 for denovolyzeR). The FWER significance threshold was set as *p*_dnEnrich_ < 3.64E−07, which was calculated by 0.05/(19,618*7) and corrected by the Bonferroni method for 19,618 genes (the larger number of genes in two models) in seven tests performed (dnLGD, dnMIS, dnMIS30, and dnALT variants in CH model, and dnLGD, dnMIS, and dnALT variants in denovolyzeR).

### Phenotypic assessment

Additional de-identified clinical records were obtained with informed consent for probands with ultra-rare severe mutations where the families were available for recontact (Supplementary Data 11). Clinical data were reviewed in consultation with the corresponding clinicians regarding the patient phenotypes and by analyzing existing or published clinical reports (Supplementary Data 12–18). For *CTCF*, we clustered and translated proband phenotype data into the corresponding Human Phenotype Ontology (HPO) annotation by using the Charité Browser; phenotypic enrichment analysis was performed based on the recurrence of the specific phenotype out of the total available clinical reports according to the HPO code (Fig. 3).

### Reporting summary

Further information on research design is available in the Nature Research Reporting Summary linked to this article.

## Supplementary information

Supplementary Information

Peer review

Dataset 1

Dataset 2

Dataset 3

Dataset 4

Dataset 5

Dataset 6

Dataset 7

Dataset 8

Dataset 9

Dataset 10

Dataset 11

Dataset 12

Dataset 13

Dataset 14

Dataset 15

Dataset 16

Dataset 17

Dataset 18

Reporting Summary

## Data Availability

The smMIP sequencing data for this study can be downloaded from the NIMH Data Archive (NDA) at 10.15154/1517561 and are available to all qualified researchers upon request after data-use certification. In order to request access to broad-use and controlled-access shared data in the NIMH Data Archive (NDA), a requester must first be affiliated with an NIH-recognized research institution registered in the NIH’s electronic research administration system, eRA Commons. The requester’s institution must also have an active Federalwide Assurance (FWA). Additionally, the requester must have a research-related need to access the data and must demonstrate adherence to any consent-based data-use restrictions in requests to access Controlled Access Permission Groups. More details about requesting access to shared data in NDA are available at https://nda.nih.gov/get/access-data.html. The URLs for data presented herein are as follows: denovo-db, http://denovo-db.gs.washington.edu/denovo-db/; Exome Aggregation Consortium (ExAC), https://gnomad.broadinstitute.org/; Online Mendelian Inheritance in Man (OMIM), http://www.omim.org/; Ensembl Variant Effect Predictor (GRCh37), http://grch37.ensembl.org/Homo_sapiens/Tools/VEP/; Combined Annotation Dependent Depletion (CADD), https://cadd.gs.washington.edu/; MedGen, https://www.ncbi.nlm.nih.gov/medgen; HPO Charité Browser, https://hpo.jax.org/app/tools/hpo-browser.

## Code availability

Custom code used in this manuscript is available at https://github.com/tianyunwang/mip_paper_2020. Tools and software used in this manuscript that include code are as following: MIPgen, https://github.com/shendurelab/MIPGEN; FreeBayes, https://github.com/ekg/freebayes; denovolyzeR, https://github.com/jamesware/denovolyzeR.
