# Author Correction: Large-scale targeted sequencing identifies risk genes for neurodevelopmental disorders

**DOI:** 10.1038/s41467-020-19289-5

**Published:** 2020-10-21

**Authors:** Tianyun Wang, Kendra Hoekzema, Davide Vecchio, Huidan Wu, Arvis Sulovari, Bradley P. Coe, Madelyn A. Gillentine, Amy B. Wilfert, Luis A. Perez-Jurado, Malin Kvarnung, Yoeri Sleyp, Rachel K. Earl, Jill A. Rosenfeld, Madeleine R. Geisheker, Lin Han, Bing Du, Chris Barnett, Elizabeth Thompson, Marie Shaw, Renee Carroll, Kathryn Friend, Rachael Catford, Elizabeth E. Palmer, Xiaobing Zou, Jianjun Ou, Honghui Li, Hui Guo, Jennifer Gerdts, Emanuela Avola, Giuseppe Calabrese, Maurizio Elia, Donatella Greco, Anna Lindstrand, Ann Nordgren, Britt-Marie Anderlid, Geert Vandeweyer, Anke Van Dijck, Nathalie Van der Aa, Brooke McKenna, Miroslava Hancarova, Sarka Bendova, Marketa Havlovicova, Giovanni Malerba, Bernardo Dalla Bernardina, Pierandrea Muglia, Arie van Haeringen, Mariette J. V. Hoffer, Barbara Franke, Gerarda Cappuccio, Martin Delatycki, Paul J. Lockhart, Melanie A. Manning, Pengfei Liu, Ingrid E. Scheffer, Nicola Brunetti-Pierri, Nanda Rommelse, David G. Amaral, Gijs W. E. Santen, Elisabetta Trabetti, Zdeněk Sedláček, Jacob J. Michaelson, Karen Pierce, Eric Courchesne, R. Frank Kooy, The SPARK Consortium, John Acampado, Andrea J. Ace, Alpha Amatya, Irina Astrovskaya, Asif Bashar, Elizabeth Brooks, Martin E. Butler, Lindsey A. Cartner, Wubin Chin, Wendy K. Chung, Amy M. Daniels, Pamela Feliciano, Chris Fleisch, Swami Ganesan, William Jensen, Alex E. Lash, Richard Marini, Vincent J. Myers, Eirene O’Connor, Chris Rigby, Beverly E. Robertson, Neelay Shah, Swapnil Shah, Emily Singer, LeeAnne G. Snyder, Alexandra N. Stephens, Jennifer Tjernagel, Brianna M. Vernoia, Natalia Volfovsky, Loran Casey White, Alexander Hsieh, Yufeng Shen, Xueya Zhou, Tychele N. Turner, Ethan Bahl, Taylor R. Thomas, Leo Brueggeman, Tanner Koomar, Jacob J. Michaelson, Brian J. O’Roak, Rebecca A. Barnard, Richard A. Gibbs, Donna Muzny, Aniko Sabo, Kelli L. Baalman Ahmed, Evan E. Eichler, Matthew Siegel, Leonard Abbeduto, David G. Amaral, Brittani A. Hilscher, Deana Li, Kaitlin Smith, Samantha Thompson, Charles Albright, Eric M. Butter, Sara Eldred, Nathan Hanna, Mark Jones, Daniel Lee Coury, Jessica Scherr, Taylor Pifher, Erin Roby, Brandy Dennis, Lorrin Higgins, Melissa Brown, Michael Alessandri, Anibal Gutierrez, Melissa N. Hale, Lynette M. Herbert, Hoa Lam Schneider, Giancarla David, Robert D. Annett, Dustin E. Sarver, Ivette Arriaga, Alexies Camba, Amanda C. Gulsrud, Monica Haley, James T. McCracken, Sophia Sandhu, Maira Tafolla, Wha S. Yang, Laura A. Carpenter, Catherine C. Bradley, Frampton Gwynette, Patricia Manning, Rebecca Shaffer, Carrie Thomas, Raphael A. Bernier, Emily A. Fox, Jennifer A. Gerdts, Micah Pepper, Theodore Ho, Daniel Cho, Joseph Piven, Holly Lechniak, Latha V. Soorya, Rachel Gordon, Allison Wainer, Lisa Yeh, Cesar Ochoa-Lubinoff, Nicole Russo, Elizabeth Berry-Kravis, Stephanie Booker, Craig A. Erickson, Lisa M. Prock, Katherine G. Pawlowski, Emily T. Matthews, Stephanie J. Brewster, Margaret A. Hojlo, Evi Abada, Elena Lamarche, Tianyun Wang, Shwetha C. Murali, William T. Harvey, Hannah E. Kaplan, Karen L. Pierce, Lindsey DeMarco, Susannah Horner, Juhi Pandey, Samantha Plate, Mustafa Sahin, Katherine D. Riley, Erin Carmody, Julia Constantini, Amy Esler, Ali Fatemi, Hanna Hutter, Rebecca J. Landa, Alexander P. McKenzie, Jason Neely, Vini Singh, Bonnie Van Metre, Ericka L. Wodka, Eric J. Fombonne, Lark Y. Huang-Storms, Lillian D. Pacheco, Sarah A. Mastel, Leigh A. Coppola, Sunday Francis, Andrea Jarrett, Suma Jacob, Natasha Lillie, Jaclyn Gunderson, Dalia Istephanous, Laura Simon, Ori Wasserberg, Angela L. Rachubinski, Cordelia R. Rosenberg, Stephen M. Kanne, Amanda D. Shocklee, Nicole Takahashi, Shelby L. Bridwell, Rebecca L. Klimczac, Melissa A. Mahurin, Hannah E. Cotrell, Cortaiga A. Grant, Samantha G. Hunter, Christa Lese Martin, Cora M. Taylor, Lauren K. Walsh, Katherine A. Dent, Andrew Mason, Anthony Sziklay, Christopher J. Smith, Magnus Nordenskjöld, Corrado Romano, Hilde Peeters, Raphael A. Bernier, Jozef Gecz, Kun Xia, Evan E. Eichler

**Affiliations:** 1 Department of Genome Sciences, University of Washington, Seattle, WA USA; 2 Rare Disease and Medical Genetics, Academic Department of Pediatrics, Bambino Gesù Children’s Hospital, Rome, Italy; 3 Genetics and Rare Diseases Research Division, Bambino Gesù Children’s Hospital, Rome, Italy; 4 Center for Medical Genetics & Hunan Provincial Key Laboratory of Medical Genetics, School of Life Sciences, Central South University, Changsha, Hunan China; 5 Paediatric and Reproductive Genetics unit, Women’s and Children’s Hospital, Adelaide, SA Australia; 6 South Australian Health and Medical Research Institute, Adelaide, SA Australia; 7 Genetics Unit, Universitat Pompeu Fabra, Hospital del Mar Research Institute (IMIM) and CIBERER, Barcelona, Spain; 8 Department of Molecular Medicine and Surgery, Center for Molecular Medicine, Karolinska Institutet, Stockholm, Sweden; 9 Department of Clinical Genetics, Karolinska University Hospital, Stockholm, Sweden; 10 Centre for Human Genetics, KU Leuven and Leuven Autism Research (LAuRes), Leuven, Belgium; 11 Department of Psychiatry and Behavioral Sciences, University of Washington, Seattle, WA USA; 12 Department of Molecular & Human Genetics, Baylor College of Medicine, Houston, TX USA; 13 Baylor Genetics, Houston, TX USA; 14 Adelaide Medical School and the Robinson Research Institute, the University of Adelaide, Adelaide, SA Australia; 15 Genetics and Molecular Pathology, SA Pathology, Adelaide, SA Australia; 16 Genetics of Learning Disability Service, Hunter New England Health Service, Waratah, NSW Australia; 17 School of Women’s and Children’s Health, University of New South Wales, Randwick, NSW Australia; 18 Children Development Behavior Center, The Third Affiliated Hospital, Sun Yat-Sen University, Guangzhou, Guangdong, China; 19 Mental Health Institute of the Second Xiangya Hospital, Central South University, Changsha, China; 20 Key Laboratory of Developmental Disorders in Children, Liuzhou Maternity and Child Healthcare Hospital, Liuzhou, China; 21 Oasi Research Institute-IRCCS, Troina, Italy; 22 Department of Medical Genetics, University of Antwerp, Antwerp, Belgium; 23 Department of Psychology, Emory University, Atlanta, GA USA; 24 Department of Biology and Medical Genetics, Charles University 2nd Faculty of Medicine and University Hospital Motol, Prague, Czech Republic; 25 Department of Neurosciences, Biomedicine and Movement Sciences, University of Verona, Verona, Italy; 26 Child Neuropsychiatry Unit, AOUI, Verona, Italy; 27 UCB Pharma, Bruxelles, Belgium; 28 Department of Clinical Genetics, Leiden University Medical Center (LUMC), Leiden, Netherlands; 29 Department of Human Genetics, Donders Institute for Brain, Cognition and Behaviour, Radboud University Medical Center, Nijmegen, Netherlands; 30 Department of Psychiatry, Donders Institute for Brain, Cognition and Behaviour, Radboud University Medical Center, Nijmegen, Netherlands; 31 Department of Translational Medicine, Federico II University, Naples, Italy; 32 Telethon Institute of Genetics and Medicine, Pozzuoli, Naples, Italy; 33 Murdoch Children’s Research Institute, Melbourne, Australia; 34 Department of Paediatrics, University of Melbourne, Parkville, VIC Australia; 35 Division of Medical Genetics, Department of Pediatrics, Stanford University, Stanford, CA USA; 36 Department of Pathology, Stanford University, Stanford, CA USA; 37 Department of Paediatrics, University of Melbourne, Royal Children’s Hospital, Melbourne, VIC Australia; 38 Department of Medicine, University of Melbourne, Austin Health, Melbourne, Australia; 39 The Florey Institute of Neuroscience and Mental Health, Parkville, VIC Australia; 40 Karakter Child and Adolescent Psychiatry Center, Nijmegen, Netherlands; 41 Department of Psychiatry and Behavioral Sciences and the MIND Institute, University of California, Davis, Sacramento, CA USA; 42 Department of Psychiatry, University of Iowa Carver College of Medicine, Iowa City, IA USA; 43 Department of Neurosciences, UC San Diego Autism Center, School of Medicine, University of California San Diego, La Jolla, CA USA; 44 CAS Center for Excellence in Brain Science and Intelligences Technology (CEBSIT), Chinese Academy of Sciences, Shanghai, China; 45 Howard Hughes Medical Institute, University of Washington, Seattle, WA USA; 46 Simons Foundation, New York, NY USA; 47 Columbia University, New York, NY USA; 48 Washington University School of Medicine, St. Louis, MO USA; 49 University of Iowa Carver College of Medicine, Iowa City, IA USA; 50 Oregon Health & Science University, Portland, OR USA; 51 Baylor College of Medicine, Houston, TX USA; 52 University of Washington School of Medicine & Howard Hughes Medical Institute, Seattle, WA USA; 53 Maine Medical Center Research Institute, Portland, OR USA; 54 University of California, Davis, Sacramento, CA USA; 55 Nationwide Children’s Hospital, Columbus, OH USA; 56 University of Miami, Coral Gables, FL USA; 57 University of Mississippi Medical Center, Jackson, MS USA; 58 University of California, Los Angeles, Los Angeles, CA USA; 59 Medical University of Southern Carolina (MUSC), Portland, OR USA; 60 Cincinnati Children’s Hospital Medical Center-Research Foundation, Cincinnati, OH USA; 61 Seattle Children’s Autism Center/UW, Seattle, WA USA; 62 University of North Carolina at Chapel Hill, Chapel Hill, NC USA; 63 Department of Child & Adolescent Psychiatry, Rush University Medical Center, Chicago, IL USA; 64 Department of Developmental & Behavioral Pediatrics, Rush University Medical Center, Chicago, IL USA; 65 Department of Neurological Sciences, Department of Pediatrics, Department of Biochemistry, Rush University Medical Center, Chicago, IL USA; 66 Cincinnati Children’s Hospital Medical Center - Research Foundation, Cincinnati, OH USA; 67 Boston Children’s Hospital (BCH), Boston, MA USA; 68 University of North Carolina at Chapel Hill, Chapel Hill, NC USA; 69 University of Washington School of Medicine, Seattle, WA USA; 70 University of California, San Diego, School of Medicine, La Jolla, CA USA; 71 Children’s Hospital of Philadelphia, Philadelphia, PA USA; 72 Boston Children’s Hospital (BCH), Boston, MA USA; 73 University of Minnesota, Minneapolis, MN USA; 74 Kennedy Krieger Institute, Baltimore, MD USA; 75 Oregon Health & Science University, Portland, OR USA; 76 University of Minnesota, Minneapolis, MN USA; 77 University of Colorado School of Medicine, Aurora, CO USA; 78 Department of Health Psychology, University of Missouri, Columbia, SC USA; 79 Thompson Center for Autism and Neurodevelopmental Disorders, University of Missouri, Columbia, SC USA; 80 Geisinger Autism & Developmental Medicine Institute, Lewisburg, PA USA; 81 Southwest Autism Research and Resource Center, Phoenix, AZ USA

**Keywords:** Mutation, Neurodevelopmental disorders, Autism spectrum disorders, Next-generation sequencing

Correction to: *Nature Communications* 10.1038/s41467-020-18723-y, published online 1 October 2020.

The original version of this Article contained an error on page 5 of the Results section, which incorrectly read ‘They are characterized by craniofacial dysmorphisms (9/10), thin vermillion border and lips (4/7), and feeding difficulties (6/11), and exhibit neonatal hypotonia (10/7)’. The correct version states ‘They are characterized by craniofacial dysmorphisms (9/10), thin vermillion border and lips (4/7), and feeding difficulties (6/11), and exhibit neonatal hypotonia (7/10)’.

