# Supplementary material for: Large-scale targeted sequencing identifies risk genes for neurodevelopmental disorders
Source: Nat Commun. 2020 Oct 1;11:4932. doi: 10.1038/s41467-020-18723-y (PMC7530681; doi:10.1038/s41467-020-18723-y)
Supplement: Supplementary file 1 — Supplementary Information [file 41467_2020_18723_MOESM1_ESM.pdf]

Large-scale targeted sequencing identifies risk genes for neurodevelopmental disorders

Wang et al.

Supplementary Information

Supplementary Information includes Supplementary Figures 1-4, Supplementary Table 1, and Supplementary Data 1-18.

Supplementary Figures

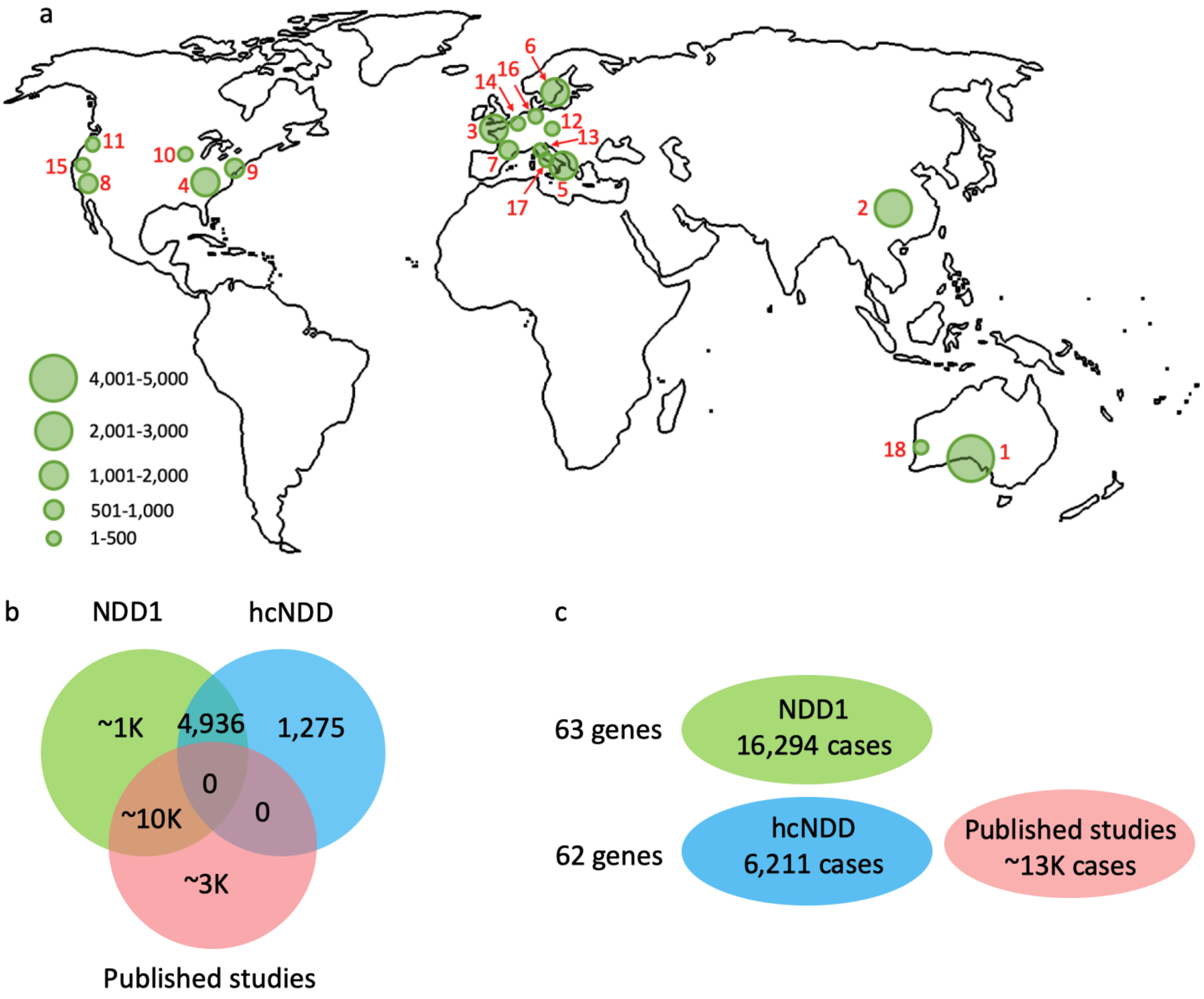

Supplementary Figure 1. Samples and genes sequenced in this study. All samples sequenced in this study using smMIPs are from the ASID network. a) Probands (n > 18K) with a primary diagnosis of ASD, DD, or ID were collected from 18 international cohorts. Circle size corresponds to the number of samples from each cohort; red numbers correspond to the cohort number in Supplementary Table 1. b) The numbers (after QC) differ slightly depending on the number of genes and therefore we indicate with an approximation sign (~). Sample overlap is indicated for three designs: NDD1 (63 genes) represents a design targeting 63 genes that were not yet established as high confidence; hcNDD (62 genes) represents a design targeting genes many of which were already known; the third portion of the Venn represents previous published smMIP studies, where variants from 62 genes in hcNDD were retrieved for a combined analysis. c) The 63 genes in panel NDD1 were screened in largest number of 16,294 NDD patients, while the 62 genes in hcNDD were screened only in 6,211 NDD cases where they had not been screened before, and the same category of variants were retrieved from ~13K NDD cases (precise number of cases may different for each gene) for the same 62 genes in hcNDD.

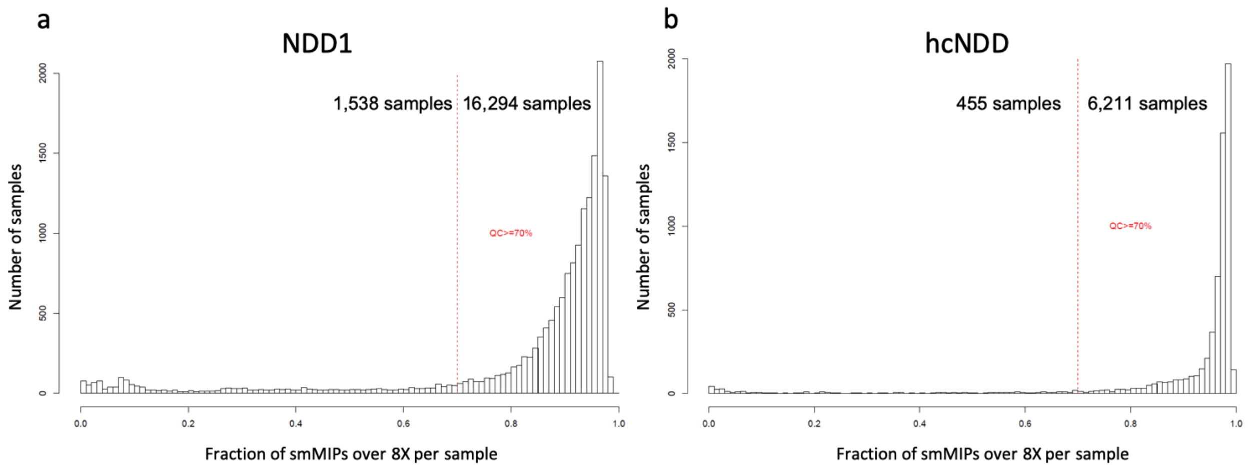

Supplementary Figure 2. QC of samples in NDD1 and hcNDD. The histogram shows the fraction of smMIPs with a read depth of over 8X per individual sample. There are 1,538 samples in NDD1 (a) and 455 samples in hcNDD (b). Less than 70% of smMIPs with a read depth over 8X failed QC and were removed from downstream analyses.

a

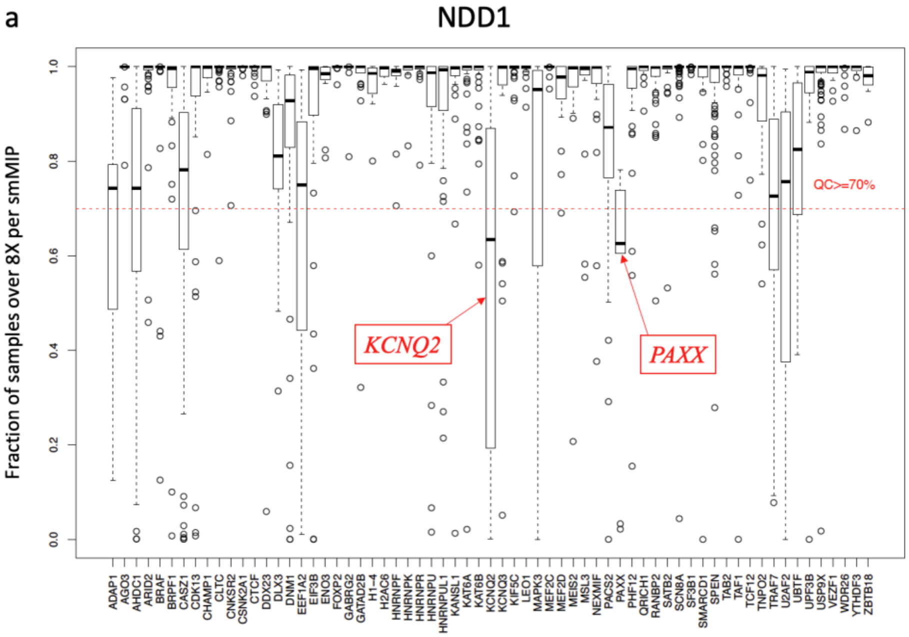

b

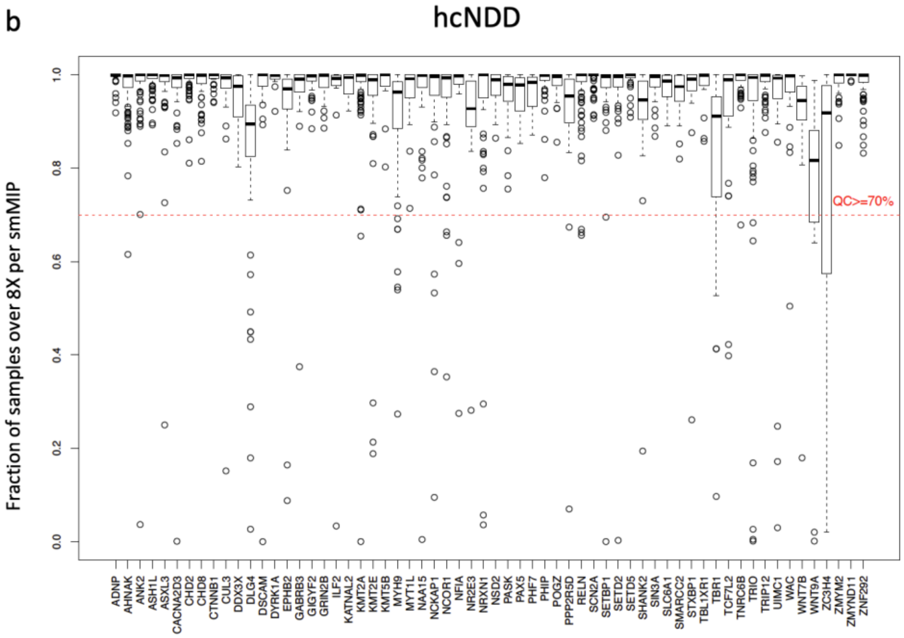

Supplementary Figure 3. QC of genes in NDD1 and hcNDD. Box and whisker plots show the fraction of samples with target bases at 8X or greater coverage for genes in NDD1 with 65 genes sequenced in 16,294 samples (after QC) (a) and hcNDD with 62 genes sequenced in 6,211 samples (after QC) (b). Two genes (*KCNQ2* and *PAXX*) in NDD1 failed QC and were removed from downstream analyses. For the box plots, the lower whisker indicates the lowest data point excluding outliers (minima), the upper whisker indicates the largest data point excluding outliers (maxima), the lower bound indicates the first quartile which is the median of the lower half of the dataset (25th percentile), the upper bound indicates the third quartile which is the median of the upper half of the dataset (75th percentile), and with the middle value of the dataset (50th percentile) indicates in the middle.

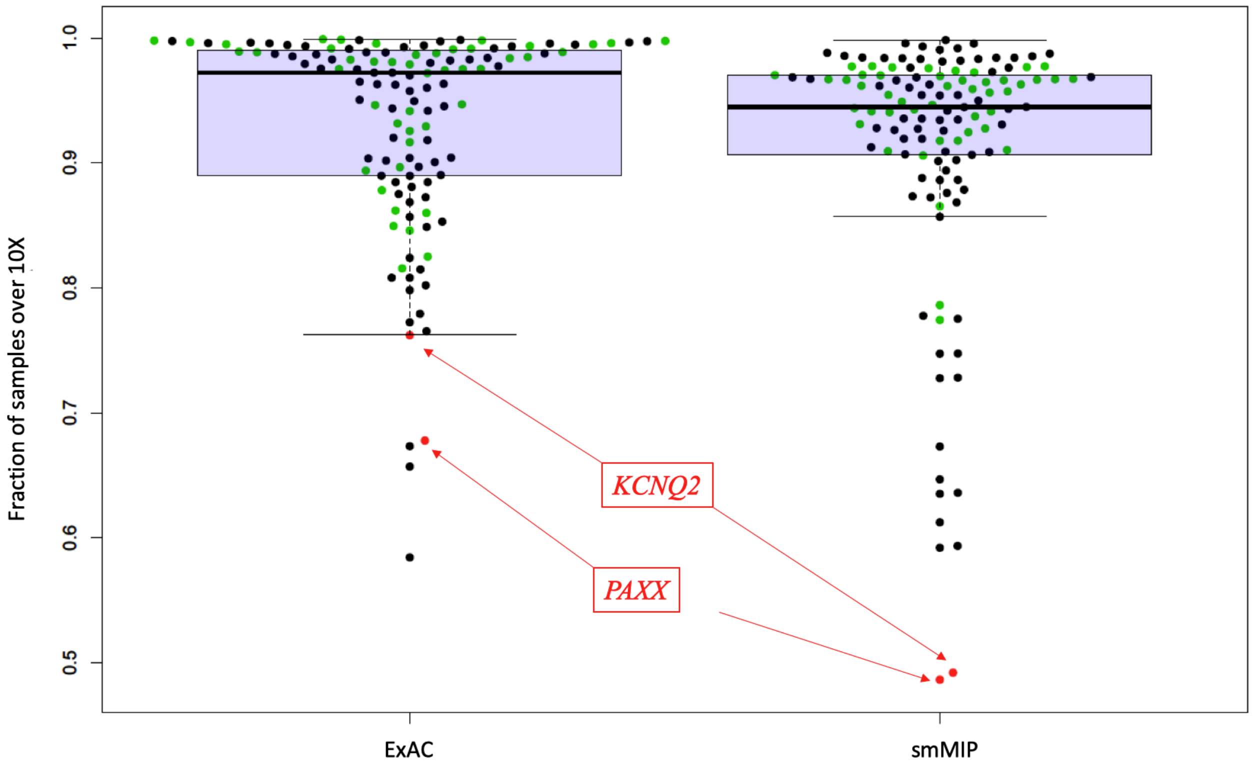

Supplementary Figure 4. Fraction of samples over 10X in ExAC and smMIP data. The fraction of samples with ≥10X read depth for ExAC (now available at https://gnomad.broadinstitute.org/) was retrieved for the same capture region as in smMIP sequencing. The average fraction was calculated per gene and plotted as the average fraction (by gene) of samples with ≥10X coverage in ExAC and smMIP data. Each dot represents a gene: green dots indicate the 48 genes at FDR significance in mutation burden analysis, and red dots indicate the two genes (*KCNQ2* and *PAXX*) that failed QC and were excluded from downstream analyses. For the box plots, the lower whisker indicates the lowest data point excluding outliers (minima), the upper whisker indicates the largest data point excluding outliers (maxima), the lower bound indicates the first quartile which is the median of the lower half of the dataset (25th percentile), the upper bound indicates the third quartile which is the median of the upper half of the dataset (75th percentile), and with the middle value of the dataset (50th percentile) indicates in the middle.

Supplementary Table 1. ASID cohorts and smMIP panels sequenced in this study.

Cohort# on Figure 1a	Cohort	Location	PI	Cohort_ID	Primary diagnosis	NDD1			hcNDD		
						All	QC failed	Post QC	All	QC failed	Post QC
1	Adelaide	Adelaide, Australia	Jozef Gecz	Adelaide1, Adelaide2	DD	2,206	161	2,045	-	-	-
				Adelaide3	DD	1,440	74	1,366	1,440	105	1,335
				Adelaide4	DD	839	41	798	839	47	792
2	ACGC	Changsha, China	Kun Xia	ACGC	ASD	2,829	317	2,512	-	-	-
3	Leuven	Leuven, Belgium	Hilde Peeters	Leuven1	ASD	904	24	880	864	11	853
				Leuven2	ASD	988	7	981	988	56	932
4	AGRE	Seattle, USA	Raphael A Bernier	AGRE	ASD	1,662	52	1,610	-	-	-
5	Troina	Troina, Italy	Corrado Romano	Troina1, Troina2	DD	1,175	153	1,022	-	-	-
				Troina3, Troina4	DD	441	42	399	441	49	392
6	Karolinska	Karolinska, Sweden	Magnus Nordenskjöld	Swedish	DD	1,499	142	1,357	-	-	-
7	Antwerp	Antwerp, Belgium	R Frank Kooy	Antwerp	DD	900	38	862	-	-	-
8	San Diego	San Diego, USA	Eric Courchesne	SanDiego1	ASD	488	6	482	-	-	-
				SanDiego2	ASD	404	0	404	404	0	404
9	TASC	Seattle, USA	Raphael A Bernier	TASC	ASD	737	203	534	-	-	-
10	Iowa	Iowa, USA	Jacob J Michaelson	Iowa	ASD	472	4	468	472	76	396
11	SAGE	Seattle, USA	Raphael A Bernier	SAGE	ASD	-	-	-	388	27	361
12	Charles	Prague, Czech Republic	Zdenek Sedlacek	Czech	ID	384	93	291	384	55	329
13	ITAN	Verona, Italy	Elisabetta Trabetti	ITAN	ASD	-	-	-	248	26	222
14	Leiden	Leiden, Netherlands	Gijs W.E. Santen	Leiden	DD	210	10	200	-	-	-
15	Autism Phenome Project	Davis, USA	David G Amaral	APP	ASD	198	132	66	-	-	-
16	Radboudumc	Nijmegen, Netherlands	Nanda Rommelse	Radboud	ASD	-	-	-	112	0	112
17	Naples	Naples, Italy	Nicola Brunetti-Pierri	Naples	ASD	-	-	-	86	3	83
18	Melbourne	Melbourne, Australia	Ingrid E Scheffer	Melbourne2	ASD	56	39	17	-	-	-
					**Total**	17,832	1,538	16,294	6,666	455	6,211

Supplementary References

1. Gregor, A. *et al.* De novo mutations in the genome organizer CTCF cause intellectual disability. *Am J Hum Genet* **93**, 124-31 (2013).
2. Hori, I. *et al.* CTCF deletion syndrome: clinical features and epigenetic delineation. *J Med Genet* **54**, 836-842 (2017).
3. Bastaki, F. *et al.* Identification of a novel CTCF mutation responsible for syndromic intellectual disability - a case report. *BMC Med Genet* **18**, 68 (2017).
4. Iossifov, I. *et al.* The contribution of de novo coding mutations to autism spectrum disorder. *Nature* **515**, 216-21 (2014).
5. Willsey, A.J. *et al.* De Novo Coding Variants Are Strongly Associated with Tourette Disorder. *Neuron* **94**, 486-499 e9 (2017).
6. Deciphering Developmental Disorders, S. Prevalence and architecture of de novo mutations in developmental disorders. *Nature* **542**, 433-438 (2017).
7. Chen, F. *et al.* Three additional de novo CTCF mutations in Chinese patients help to define an emerging neurodevelopmental disorder. *American Journal of Medical Genetics Part C-Seminars in Medical Genetics* **181**, 218-225 (2019).
8. Konrad, E.D.H. *et al.* CTCF variants in 39individuals with a variable neurodevelopmental disorder broaden the mutational andclinical spectrum. *Genetics In Medicine* (2019).
9. Epi, K.C. *et al.* De novo mutations in epileptic encephalopathies. *Nature* **501**, 217-21 (2013).
10. Carvill, G.L. *et al.* Targeted resequencing in epileptic encephalopathies identifies de novo mutations in CHD2 and SYNGAP1. *Nat Genet* **45**, 825-30 (2013).
11. Hamdan, F.F. *et al.* De novo mutations in moderate or severe intellectual disability. *PLoS Genet* **10**, e1004772 (2014).
12. de Kovel, C.G. *et al.* Targeted sequencing of 351 candidate genes for epileptic encephalopathy in a large cohort of patients. *Mol Genet Genomic Med* **4**, 568-80 (2016).
13. Leduc, M.S. *et al.* Clinical and molecular characterization of de novo loss of function variants in HNRNPU. *Am J Med Genet A* **173**, 2680-2689 (2017).
14. Lim, E.T. *et al.* Rates, distribution and implications of postzygotic mosaic mutations in autism spectrum disorder. *Nat Neurosci* **20**, 1217-1224 (2017).
15. Yates, T.M. *et al.* De novo mutations in HNRNPU result in a neurodevelopmental syndrome. *Am J Med Genet A* **173**, 3003-3012 (2017).
16. Depienne, C. *et al.* Genetic and phenotypic dissection of 1q43q44 microdeletion syndrome and neurodevelopmental phenotypes associated with mutations in ZBTB18 and HNRNPU. *Hum Genet* **136**, 463-479 (2017).
17. Bramswig, N.C. *et al.* Heterozygous HNRNPU variants cause early onset epilepsy and severe intellectual disability. *Hum Genet* **136**, 821-834 (2017).
18. Shimada, S. *et al.* An episode of acute encephalopathy with biphasic seizures and late reduced diffusion followed by hemiplegia and intractable epilepsy observed in a patient with a novel frameshift mutation in HNRNPU. *Brain Dev* **40**, 813-818 (2018).
19. Rauch, A. *et al.* Range of genetic mutations associated with severe non-syndromic sporadic intellectual disability: an exome sequencing study. *Lancet* **380**, 1674-82 (2012).
20. Bosch, D.G. *et al.* Novel genetic causes for cerebral visual impairment. *Eur J Hum Genet* **24**, 660-5 (2016).
21. Miceli, F. *et al.* Early-onset epileptic encephalopathy caused by gain-of-function mutations in the voltage sensor of Kv7.2 and Kv7.3 potassium channel subunits. *J Neurosci* **35**, 3782-93 (2015).

22. Maljevic, S. *et al.* Novel KCNQ3 Mutation in a Large Family with Benign Familial Neonatal Epilepsy: A Rare Cause of Neonatal Seizures. *Mol Syndromol* **7**, 189-196 (2016).
23. Neubauer, B.A. *et al.* KCNQ2 and KCNQ3 mutations contribute to different idiopathic epilepsy syndromes. *Neurology* **71**, 177-83 (2008).
24. Hahn, A. & Neubauer, B.A. Sodium and potassium channel dysfunctions in rare and common idiopathic epilepsy syndromes. *Brain Dev* **31**, 515-20 (2009).
25. Singh, N.A. *et al.* KCNQ2 and KCNQ3 potassium channel genes in benign familial neonatal convulsions: expansion of the functional and mutation spectrum. *Brain* **126**, 2726-37 (2003).
26. Sands, T.T. *et al.* Rapid and safe response to low-dose carbamazepine in neonatal epilepsy. *Epilepsia* **57**, 2019-2030 (2016).
27. Hirose, S. *et al.* A novel mutation of KCNQ3 (c.925T-->C) in a Japanese family with benign familial neonatal convulsions. *Ann Neurol* **47**, 822-6 (2000).
28. Uehara, A. *et al.* Altered KCNQ3 potassium channel function caused by the W309R pore-helix mutation found in human epilepsy. *J Membr Biol* **222**, 55-63 (2008).
29. Sugiura, Y. *et al.* Lack of potassium current in W309R mutant KCNQ3 channel causing benign familial neonatal convulsions (BFNC). *Epilepsy Res* **84**, 82-5 (2009).
30. Charlier, C. *et al.* A pore mutation in a novel KQT-like potassium channel gene in an idiopathic epilepsy family. *Nat Genet* **18**, 53-5 (1998).
31. Soldovieri, M.V. *et al.* Novel KCNQ2 and KCNQ3 mutations in a large cohort of families with benign neonatal epilepsy: first evidence for an altered channel regulation by syntaxin-1A. *Hum Mutat* **35**, 356-67 (2014).
32. Li, H.Y. *et al.* [Clinical and mutational analysis of KCNQ3 gene in a Chinese family with benign familial neonatal convulsions]. *Zhonghua Yi Xue Yi Chuan Xue Za Zhi* **23**, 374-7 (2006).
33. Li, H. *et al.* A novel mutation of KCNQ3 gene in a Chinese family with benign familial neonatal convulsions. *Epilepsy Res* **79**, 1-5 (2008).
34. Fister, P., Soltirovska-Salamon, A., Debeljak, M. & Paro-Panjan, D. Benign familial neonatal convulsions caused by mutation in KCNQ3, exon 6: a European case. *Eur J Paediatr Neurol* **17**, 308-10 (2013).
35. Miceli, F. *et al.* A novel KCNQ3 mutation in familial epilepsy with focal seizures and intellectual disability. *Epilepsia* **56**, e15-20 (2015).
36. Allen, N.M. *et al.* The variable phenotypes of KCNQ-related epilepsy. *Epilepsia* **55**, e99-105 (2014).
37. Grinton, B.E. *et al.* Familial neonatal seizures in 36 families: Clinical and genetic features correlate with outcome. *Epilepsia* **56**, 1071-80 (2015).
38. Fusco, C., Frattini, D. & Bassi, M.T. A novel KCNQ3 gene mutation in a child with infantile convulsions and partial epilepsy with centrotemporal spikes. *Eur J Paediatr Neurol* **19**, 102-3 (2015).
39. Miceli, F. *et al.* Neutralization of a unique, negatively-charged residue in the voltage sensor of K V 7.2 subunits in a sporadic case of benign familial neonatal seizures. *Neurobiol Dis* **34**, 501-10 (2009).
40. Lemke, J.R. *et al.* Targeted next generation sequencing as a diagnostic tool in epileptic disorders. *Epilepsia* **53**, 1387-98 (2012).
41. Gilling, M. *et al.* Dysfunction of the Heteromeric KV7.3/KV7.5 Potassium Channel is Associated with Autism Spectrum Disorders. *Front Genet* **4**, 54 (2013).

42. Zara, F. *et al.* Genetic testing in benign familial epilepsies of the first year of life: clinical and diagnostic significance. *Epilepsia* **54**, 425-36 (2013).
43. Bassi, M.T. *et al.* Functional analysis of novel KCNQ2 and KCNQ3 gene variants found in a large pedigree with benign familial neonatal convulsions (BFNC). *Neurogenetics* **6**, 185-93 (2005).
44. Cohen, J.S. *et al.* Further evidence that de novo missense and truncating variants in ZBTB18 cause intellectual disability with variable features. *Clin Genet* **91**, 697-707 (2017).
45. de Munnik, S.A. *et al.* A de novo non-sense mutation in ZBTB18 in a patient with features of the 1q43q44 microdeletion syndrome. *Eur J Hum Genet* **22**, 844-6 (2014).
46. Lopes, F. *et al.* Identification of novel genetic causes of Rett syndrome-like phenotypes. *J Med Genet* **53**, 190-9 (2016).
47. van der Schoot, V. *et al.* Toward clinical and molecular understanding of pathogenic variants in the ZBTB18 gene. *Mol Genet Genomic Med* **6**, 393-400 (2018).
48. Sharma, V.P. *et al.* Mutations in TCF12, encoding a basic helix-loop-helix partner of TWIST1, are a frequent cause of coronal craniosynostosis. *Nat Genet* **45**, 304-7 (2013).
49. di Rocco, F. *et al.* Clinical spectrum and outcomes in families with coronal synostosis and TCF12 mutations. *Eur J Hum Genet* **22**, 1413-6 (2014).
50. Paumard-Hernandez, B. *et al.* Expanding the mutation spectrum in 182 Spanish probands with craniosynostosis: identification and characterization of novel TCF12 variants. *Eur J Hum Genet* **23**, 907-14 (2015).
51. Lee, E. *et al.* A craniosynostosis massively parallel sequencing panel study in 309 Australian and New Zealand patients: findings and recommendations. *Genet Med* **20**, 1061-1068 (2018).
52. Goos, J.A. *et al.* Identification of Intragenic Exon Deletions and Duplication of TCF12 by Whole Genome or Targeted Sequencing as a Cause of TCF12-Related Craniosynostosis. *Hum Mutat* **37**, 732-6 (2016).
53. Timberlake, A.T. *et al.* Co-occurrence of frameshift mutations in SMAD6 and TCF12 in a child with complex craniosynostosis. *Hum Genome Var* **5**, 14 (2018).
54. Goumenos, A. *et al.* Two novel variants in the TCF12 gene identified in cases with craniosynostosis. *Appl Clin Genet* **12**, 19-25 (2019).
55. Yilmaz, E., Mihci, E., Nur, B. & Alper, O.M. Coronal craniosynostosis due to TCF12 mutations in patients from Turkey. *Am J Med Genet A* **179**, 2241-2245 (2019).
56. Yuen, R.K. *et al.* Genome-wide characteristics of de novo mutations in autism. *NPJ Genom Med* **1**, 160271-1602710 (2016).
57. De Rubeis, S. *et al.* Synaptic, transcriptional and chromatin genes disrupted in autism. *Nature* **515**, 209-15 (2014).
58. Brandler, W.M. *et al.* Paternally inherited cis-regulatory structural variants are associated with autism. *Science* **360**, 327-331 (2018).
