# Supplementary material for: Large-scale targeted sequencing identifies risk genes for neurodevelopmental disorders
Source: Nat Commun. 2020 Oct 1;11:4932. doi: 10.1038/s41467-020-18723-y (PMC7530681; doi:10.1038/s41467-020-18723-y)
Supplement: Supplementary file 21 — Reporting Summary [file 41467_2020_18723_MOESM21_ESM.pdf]

nature research

Corresponding author(s): Evan E Eichler

Last updated by author(s): Aug 15, 2020

Reporting Summary

Nature Research wishes to improve the reproducibility of the work that we publish. This form provides structure for consistency and transparency in reporting. For further information on Nature Research policies, see Authors & Referees and the Editorial Policy Checklist.

Statistics

For all statistical analyses, confirm that the following items are present in the figure legend, table legend, main text, or Methods section.

n/a	Confirmed	
☐	☒	The exact sample size (*n*) for each experimental group/condition, given as a discrete number and unit of measurement
☐	☒	A statement on whether measurements were taken from distinct samples or whether the same sample was measured repeatedly
☐	☒	The statistical test(s) used AND whether they are one- or two-sided<br>*Only common tests should be described solely by name; describe more complex techniques in the Methods section.*
☐	☒	A description of all covariates tested
☐	☒	A description of any assumptions or corrections, such as tests of normality and adjustment for multiple comparisons
☐	☒	A full description of the statistical parameters including central tendency (e.g. means) or other basic estimates (e.g. regression coefficient) AND variation (e.g. standard deviation) or associated estimates of uncertainty (e.g. confidence intervals)
☐	☒	For null hypothesis testing, the test statistic (e.g. *F*, *t*, *r*) with confidence intervals, effect sizes, degrees of freedom and *P* value noted<br>*Give P values as exact values whenever suitable.*
☒	☐	For Bayesian analysis, information on the choice of priors and Markov chain Monte Carlo settings
☒	☐	For hierarchical and complex designs, identification of the appropriate level for tests and full reporting of outcomes
☐	☒	Estimates of effect sizes (e.g. Cohen's *d*, Pearson's *r*), indicating how they were calculated

Our web collection on statistics for biologists contains articles on many of the points above.

Software and code

Policy information about availability of computer code

Data collection	No software was used for data collection.
Data analysis	R v3.6.1 (statistical analysis)<br>python v2.7.3 (run MIPgen script)<br>MIPgen v1.1-14-gf953b0b (smMIP design and smMIP data analysis)<br>pear v0.9.5 (Pair-end read merge)<br>BWA v0.7.15-r1140 (sequence mapping)<br>SAMtools v1.9 (sequence alignment)<br>FreeBayes v1.0.2-6-g3ce827d (variant calling)<br>GATK v3.7 (VCF combine)<br>vcflib/20160331 (VCF filtering)<br>tabix/0.2.6 (VCF index)<br>bcftools/1.4 (VCF annotate)<br>Ensembl VEP GRCh37 release 94 - October 2018 (variant annotation)<br>CADD v1.3 (CADD score annotation)<br>KING v2.2.5 (Kinship analysis)<br>denovolyzeR v0.2.0 (de novo enrichment analysis)

For manuscripts utilizing custom algorithms or software that are central to the research but not yet described in published literature, software must be made available to editors/reviewers. We strongly encourage code deposition in a community repository (e.g. GitHub). See the Nature Research guidelines for submitting code & software for further information.

Data

Policy information about availability of data

All manuscripts must include a data availability statement. This statement should provide the following information, where applicable:

- Accession codes, unique identifiers, or web links for publicly available datasets
- A list of figures that have associated raw data
- A description of any restrictions on data availability

Data availability
The smMIP sequencing data for this study can be downloaded from the NIMH Data Archive (NDA) at https://dx.doi.org/10.15154/1517561 and are available to all qualified researchers upon request after data-use certification. In order to request access to broad-use and controlled-access shared data in the NIMH Data Archive (NDA), requesters must first be affiliated with an NIH-recognized research institution registered in the NIH's electronic research administration system, eRA Commons. The requester's institution must also have an active Federalwide Assurance (FWA). Additionally, requester must have a research-related need to access the data and must demonstrate adherence to any consent-based data use restrictions in requests to access Controlled Access Permission Groups. More details about requesting access to shared data in NDA are available at https://nda.nih.gov/get/access-data.html. The URLs for data presented herein are as follows: denovo-db, http://denovo-db.gs.washington.edu/denovo-db/; Exome Aggregation Consortium (ExAC), https://gnomad.broadinstitute.org/; Online Mendelian Inheritance in Man (OMIM), http://www.omim.org/; Ensembl Variant Effect Predictor (GRCh37), http://grch37.ensembl.org/Homo_sapiens/Tools/VEP/; Combined Annotation Dependent Depletion (CADD), https://cadd.gs.washington.edu/; MedGen, https://www.ncbi.nlm.nih.gov/medgen; HPO Charité Browser, https://hpo.jax.org/app/tools/hpo-browser.

Code availability
Custom code used in this manuscript is available at https://github.com/tianyunwang/mip_paper_2020. Tools and software used in this manuscript that include code are as following: MIPgen, https://github.com/shendurelab/MIPGEN; FreeBayes, https://github.com/ekg/freebayes; denovolyzeR, https://github.com/jamesware/denovolyzeR.

Field-specific reporting

Please select the one below that is the best fit for your research. If you are not sure, read the appropriate sections before making your selection.

☒ Life sciences ☐ Behavioural & social sciences ☐ Ecological, evolutionary & environmental sciences

For a reference copy of the document with all sections, see nature.com/documents/nr-reporting-summary-flat.pdf

Life sciences study design

All studies must disclose on these points even when the disclosure is negative.

Sample size	We obtained the maximum possible number of NDD samples with DNA available of good quality from the ASID network and required that samples had not been sequenced previously for the 125 genes for inclusion in this study. There were no statistical methods used to predetermine the sample size in this study. With the current sample size (n > 16,000), we are still underpowered to establish the mutational burden of ultra-rare (MAF < 0.01%) LGD and MIS30 variants for all 125 genes. Nevertheless, this was sufficient to identify significance by standard case–control criteria for 48 genes (FDR 5%), of which 6 genes reach an even more stringent significance level (FWER).
Data exclusions	There were 1,538 samples (784 ASD and 754 DD/ID) and two genes (KCNQ2 and PAXX) from smMIP pool NDD1 and 455 DNA samples (199 ASD and 256 DD/ID) in smMIP pool hcNDD that failed QC based on read-depth coverage statistics (Supplementary Figure 2, Supplementary Figure 3); these samples and genes were removed from subsequent downstream analyses. More details are described in the Results and Methods sections.
Replication	The combination of 10,927 NDD trios in denovo-db (v1.5) and 6,499 new ASD trios from SPARK-27K release were used as a "discovery" cohort (n=17,426) for de novo enrichment analyses; then a larger sample set with samples from the SPARK pilot study, the DDD study, and the ASC study integrated was used as a "replication" cohort (n=48,281). The genes with significant excess of DNM identified in the "discovery" cohort were successfully replicated in the "replication" cohort, and four more significant genes were identified.
Randomization	ASID samples and genes were allocated into two targeted sequencing panels based on whether the genes and samples were previously (panel NDD1) or not previously (panel hcNDD) sequenced. In NDD1, we sequenced 63 genes in 16,294 NDD cases (after QC); and in hcNDD, we sequenced 62 genes in 6,211 NDD cases (after QC). We also retrieved the same categories of ultra-rare variants for the 62 genes in hcNDD from our published smMIP studies on a subset of ASID samples. Data were then combined together for downstream analyses, and there was no randomization experiments.
Blinding	As described above, genes and samples were allocated into two panels/groups simply based on whether they had been sequenced previously or not. All sequenced variants, including those retrieved from published studies, were combined together as one group for downstream analyses. Affected status is needed to perform the statistical analyses, and therefore none of the samples' status was blinded.

Reporting for specific materials, systems and methods

We require information from authors about some types of materials, experimental systems and methods used in many studies. Here, indicate whether each material, system or method listed is relevant to your study. If you are not sure if a list item applies to your research, read the appropriate section before selecting a response.

Materials & experimental systems

n/a	Involved in the study
☒	☐ Antibodies
☒	☐ Eukaryotic cell lines
☒	☐ Palaeontology
☒	☐ Animals and other organisms
☐	☒ Human research participants
☒	☐ Clinical data

Methods

n/a	Involved in the study
☒	☐ ChIP-seq
☒	☐ Flow cytometry
☒	☐ MRI-based neuroimaging

Human research participants

Policy information about studies involving human research participants

Population characteristics	Patient samples of both genders with a primary diagnosis of ASD or DD/ID were obtained from the international Autism Spectrum/Intellectual Disability (ASID) network, which includes 18 centers around the world. Samples selected in this study had no exome nor genome sequenced previously (Supplementary Figure 1, Supplementary Table 1). Samples are composed of multiple ethnicities with the majority from EUR population. More details are described in Methods section.
Recruitment	ASID is an international consortium that has expanded to include 18 centers around the world with over 20,000 NDD patients recruited. The diagnostic criteria across different recruiting sites may slightly vary to each other; some centers are mainly focused on recruiting ASD cases while the others may focus on recruiting DD/ID cases instead or both. All participants recruited across those centers together form a population with different ethnicities, although with the majority are from EUR population. These biases may lead to different mutation spectrum of each subcohort.
Ethics oversight	All targeted sequencing, Sanger variant validation, transmission analysis, and clinical recontact performed on the individuals in this study were approved by the University of Washington Institutional Review Board (IRB), in accordance with the ethical standards of the responsible local institutional and national committees with informed consent from the participants or their parents. De-identified clinical records were obtained with informed consent from probands with ultra-rare severe mutations where the families were available for recontact (Supplementary Data 11).

Note that full information on the approval of the study protocol must also be provided in the manuscript.
