# Supplementary material for: Large-scale targeted sequencing identifies risk genes for neurodevelopmental disorders
Source: Nat Commun. 2020 Oct 1;11:4932. doi: 10.1038/s41467-020-18723-y (PMC7530681; doi:10.1038/s41467-020-18723-y)
Supplement: Supplementary file 2 — Peer review [file 41467_2020_18723_MOESM2_ESM.pdf]

REVIEWER COMMENTS</B>

Reviewer #1 (Remarks to the Author):

Authors described the evaluation of 125 genes in >16000 NDD cases compared to nonpsychiatric controls from ExAC regarding the mutation burden. They showed 48 genes showing significant burden of ultra-rare gene-disruptive mutation (FDR 5%). Authors also evaluated DNM excess in 17426 NDD trios (10924 NDD trios previously published by themselves and 6449 ASD trios from SPARK data). They identified 90 genes were enriched for DNMs (FDR 5%) and 61 genes reach the exome-wide significance among 125 genes. They could show the statistical evidence using data of a large number of patients who could not be subjected to whole exome/genome sequencing. At last, they picked up seven NDD risk genes with a number of likely mutations for the sufficient phenotype-genotype correlation from this large-scale target-sequencing study. Regarding this manuscript, authors newly sequenced 62 genes in 6666 new NDD cases (3562 ASD and 3104 ID/DD cases) whose data can be newly deposited. In tables 2, authors mentioned "novel", but many known genes were seen. Although this reviewer recognizes authors' significant efforts for this manuscript, but several concerns were raised as follows.

1. This reviewer thinks authors could find several candidate genes with statistical evidence using a large data set of smMIP sequencing study, but this is not big surprising as they initially chose anyway highly likely genes. For example, HGMD already registered 46 CTCF mutations, 80 HNRNPU mutations, 40 KCNQ3 mutations, 39 ZBTB18 mutations, 81 TCF12 mutations (mostly in craniosynostosis though), 9 SPEN mutations, and 2 LEO1 mutations. I do recognize some novelity in TCF12 and LEO1 mutations for NDD in this study. This reviewer thinks authors could successfully show their statistical approach was appropriate in delineating candidate NDD genes using approximately 16000 cases close to 20000, though.

2. To this reviewer, it is very hard to grasp how these analyzed populations of NDD were overlapped. For example, 16294 NDD cases for NDD1, and 6211 NDD cases plus ~13000 NDD cases are completely different or significantly overlapped in Figure 1. The same is true in de novo enrichment analysis. Authors should clarify these population structure of patient's groups. The authors stated that they removed sample overlap in SPARK, ASC, and DDD data at the section of "Variant annotation and validation" in Methods. How did they do it?

3. This reviewer does not know how appropriate to say exome-wide significance even in non-exome study.

4. Page 6, last line: Among 110 variants for transmission assessment, 70 inherited variants were found. How about pathogenicity of such inherited variants? Any comments are required. Or this transmission assessment was not considered in evaluation?

5. In Figure 3, which variants are novel?

6. Page 8, line 18: In reevaluation of genes for excess DNMs section, this reviewer cannot fully understand "6,499 new AASD trios from 5,911 complete families".

7. Page 10, line 21: The authors performed case-control analysis of ultra rare and damaging variants at 125 candidate genes and found that 48 genes were significantly enriched (5% FDR). The authors stated that "SPEN is newly identified in this study with a significant burden" at the section of "Genotype-phenotype correlations" while the authors didn't mention which other 47 genes were novel or not. The authors should describe what in the 47 genes were novel.

Reviewer #2 (Remarks to the Author):

The authors present data on 125 candidate de novo mutations genes for over 16,000 subjects with NDD. The authors make an excellent point in the Introduction, that, to date, very few of the reported candidate genes have been studied at sufficient depth with sufficient cases to provide genome-wide statistical significance or to establish meaningful genotype-phenotype correlations. The approach outlined here goes some distance to provide that for a subset of implicated genes, and hopefully will set precedents for the study of further candidate genes. Where exome/genome sequence can be used as the discovery tool, the approach here attempts to validate these discoveries in a meaningful way. The paper is very well written, and a valuable contribution and reference point for the research and clinical communities.

I have a few comments and suggestions for clarification:

What criteria or other considerations were used to rank and select the 125 candidate genes chosen?

Was it a trio study or just probands? It implies in the Results section that parents were tested (by referring to paternal/maternal/de novo) but I cannot find any explicit mention of this in the methods. Presumably Sanger sequencing, but this should be clearer.

Minor comments:
Page 7 line 13: delete “are”
Page 9: paragraph 2, line2-3: insert “were” after “from families that..”
Figure 3: I’m concerned that Figs 3c-h include text indicating specific mutations that will be too small to read (especially Fig3g, SPEN which is an even smaller font). I would suggest that these figures be separated from the rest of Fig 3 and moved into a new figure where they can be enlarged.

Response to referees
NCOMMS-20-14144

Reviewer #1 (Remarks to the Author):

Authors described the evaluation of 125 genes in >16000 NDD cases compared to nonpsychiatric controls from ExAC regarding the mutation burden. They showed 48 genes showing significant burden of ultra-rare gene-disruptive mutation (FDR 5%). Authors also evaluated DNM excess in 17426 NDD trios (10924 NDD trios previously published by themselves and 6449 ASD trios from SPARK data). They identified 90 genes were enriched for DNMs (FDR 5%) and 61 genes reach the exome-wide significance among 125 genes. They could show the statistical evidence using data of a large number of patients who could not be subjected to whole exome/genome sequencing. At last, they picked up seven NDD risk genes with a number of likely mutations for the sufficient phenotype-genotype correlation from this large-scale target-sequencing study. Regarding this manuscript, authors newly sequenced 62 genes in 6666 new NDD cases (3562 ASD and 3104 ID/DD cases) whose data can be newly deposited. In tables 2, authors mentioned “novel”, but many known genes were seen. Although this reviewer **recognizes authors’ significant efforts** for this manuscript, but several concerns were raised as follows.

1. This reviewer thinks authors could find several candidate genes with statistical evidence using a large data set of smMIP sequencing study, but this is not big surprising as they initially chose anyway highly likely genes. For example, HGMD already registered 46 CTCF mutations, 80 HNRNPU mutations, 40 KCNQ3 mutations, 39 ZBTB18 mutations, 81 TCF12 mutations (mostly in craniosynostosis though), 9 SPEN mutations, and 2 LEO1 mutations. I do recognize some novelty in TCF12 and LEO1 mutations for NDD in this study. This reviewer thinks authors could successfully show their statistical approach was appropriate in delineating candidate NDD genes using approximately 16000 cases close to 20000, though.

The reviewer is correct. We included in our selection some of our best candidate genes given a subset were already known and these served as a positive control for the experiments. For other genes, we successfully changed the category from likely pathogenic to pathogenic (e.g., *SPEN* and *TCF12*). Notwithstanding the large number of patients screened, some genes remain borderline significant but the evidence points to the need for larger sample testing, which is critical going forward. We believe these results are valuable to both the research and diagnostic clinical community. The high-confidence genes serve in essence as positive controls for the experiment. Clinically, for many of the known genes (arguably some of the most important for autism and developmental delay), we essentially doubled the number of cases for clinical investigation from previous studies.

As for the statistical approach, we corrected for both the number of cases (>16,000) and the number of genes (~20,000) in the statistical analyses we performed. The number of NDD cases sequenced in each gene and corrected are listed in column C of

Supplementary Table 11, ranging from 16,294 to 19,847 (after QC). These numbers were used as denominators in the mutation burden analysis for ultra-rare LGD and MIS30 variants compared with 45,635 ExAC non-psych samples as controls; also in the process of multiple test correction, we corrected for a p-value cutoff for FDR significance based on the 125 tested. In the application of FWER significance, we applied a p-value cutoff for FWER significance based on the total number of genes in the human genome (n=20,000). We describe the details of multiple test correction for statistical significance in the corresponding methods and legend sections (bold text).

Methods section:

*"**Statistical analyses.** All statistical tests were performed using the R programming language (version 3.6.1). Benjamini–Hochberg FDR or Bonferroni FWER was applied when appropriate for multiple testing correction as described in the relevant sections. For mutation burden analysis, Fisher's exact test (one-tailed) was used to compare the number of LGD and MIS30 variants from smMIP sequencing (cases) with those from the ExAC non-psych subset (controls), false positive variants by Sanger validation and variants with insufficient coverage (<90% samples with at least 10X coverage) in ExAC were excluded. **The FDR significance threshold was set as $q_{mutBurden} < 0.05$ where the q-value was corrected by Benjamini–Hochberg method for the total number of genes in this study ($n_{genes} = 125$); the FWER significance threshold was set as $p_{mutBurden} <$ 1.25E-06, which was calculated by 0.05/(20,000*2) and corrected by Bonferroni method for 20,000 genes in human genome and two tests performed (LGD and MIS30 variants).** For de novo enrichment analysis, we applied both the CH model[2] and denovolyzeR[26] methods to assess the enrichment for four classes of DNM: dnLGD, dnMIS, dnMIS30, and dnALT. We applied denovolyzeR (v0.2.0) using default settings where dnMIS30 variants are not assessed; a modified CH model[4] was applied to include the evaluation of dnMIS30 variants. Both methods apply their own underlying mutation rate estimates to generate the prior probabilities for observing a specific number and class of mutations for a given gene. Briefly, the CH model estimates the number of expected DNMs by incorporating chimpanzee–human coding sequence divergence and the length of the gene; denovolyzeR estimates mutation rates based on trinucleotide context, mutational biases such as CpG hotspots, and macaque–human gene comparisons. Default parameters were used for both methods, and the expected mutation rate of 1.8 DNMs per exome was set to the CH model as an upper bound baseline. **The FDR significance threshold was set as $q_{dnEnrich} < 0.05$ and corrected by the Benjamini–Hochberg method for the number of genes in each model (18,946 for CH model and 19,618 for denovolyzeR). The FWER significance threshold was set as $p_{dnEnrich} <$ 3.64E-07, which was calculated by 0.05/(19,618*7) and corrected by the Bonferroni method for 19,618 genes (the larger number of genes in two models) in seven tests performed (dnLGD, dnMIS, dnMIS30, and dnALT variants in CH model, and dnLGD, dnMIS, and dnALT variants in denovolyzeR).**"*

Table legend:

"Table 1. Genes with a significant burden for ultra-rare severe variants.

*Fisher's exact test (one-tailed) for LGD and MIS30 variants from smMIP sequencing compared to the ExAC (r0.3) non-psych subset identified 48 genes significant at the FDR level, of which, six genes reach FWER significance. **The FDR significance threshold $q_{mutBurden} < 0.05$ was corrected by the Benjamini–Hochberg method for 125 genes in this study; the FWER significance threshold $p_{mutBurden} <$ 1.25E-06 was corrected by the Bonferroni method for 20,000 genes in human genome and two tests performed (LGD and MIS30 variants).** *Indicates 25 genes showing new mutational burden significance in case–control analysis of ultra-rare LGD and MIS30 variants in this study. See Supplementary Table 11 for underlying data."*

*“**Table 2. Genes reaching new de novo enrichment significance.***
Five genes newly reached FDR significance and seven genes reached FWER significance in the de novo enrichment analysis, compared to Coe et al., 2019[25], using the same methods (CH model and denovolyzeR) with DNMs in 17,426 NDD trios combined from denovo-db (v1.5) and SPARK-27K. ***The FDR significance threshold $q_{dnEnrich}$ < 0.05 was corrected by the Benjamini–Hochberg method for genes in each method (18,946 genes in CH model and 19,618 genes in denovolyzeR); the FWER significance threshold $p_{dnEnrich}$ < 3.64E-07 was corrected by the Bonferroni method for 19,618 genes and seven tests (dnLGD, dnMIS, dnMIS30, and dnALT variants in CH model, and dnLGD, dnMIS, and dnALT variants in denovolyzeR).*** *Coe253 indicates whether the gene is in the 253 genes reported significant (FDR 5%) in Coe et al., 2019[25]; ASC102 indicates whether the gene is in the 102 genes reported as significant (FDR 10%) in Satterstrom et al., 2020[8]; and DDD299 indicates whether the gene is in the 299 genes reported as significant in Kaplanis et al., 2019, bioRxiv preprint. Note different methods and significant threshold were applied in those three studies. See Supplementary Table 11 for underlying data.”*

2. To this reviewer, it is very hard to grasp how these analyzed populations of NDD were overlapped. For example, 16294 NDD cases for NDD1, and 6211 NDD cases plus ~13000 NDD cases are completely different or significantly overlapped in Figure 1. The same is true in de novo enrichment analysis. Authors should clarify these population structure of patient’s groups. The authors stated that they removed sample overlap in SPARK, ASC, and DDD data at the section of "Variant annotation and validation" in Methods. How did they do it?

This is indeed complicated because experiments were performed over a period of several years with sample enrollment ongoing. To help people understand the degree of overlap among samples and study designs, we made some modifications to the former Supplementary Figure 1. First, we start with the 18 different referral sites (Supplementary Figure 1a) showing the approximate number of NDD cases that contributed to this study. We also include some simple Venn diagrams showing the sample and gene overlap in Supplementary Figure 1b-c. Here, we break down the 125 genes into two groups: high-confidence NDD genes in hcNDD set with 62 genes where we examined fewer cases (because they had been studied before in samples that collected earlier) when compared to the new NDD1 gene set with 63 genes. Numbers of samples and genes are shown after QC.

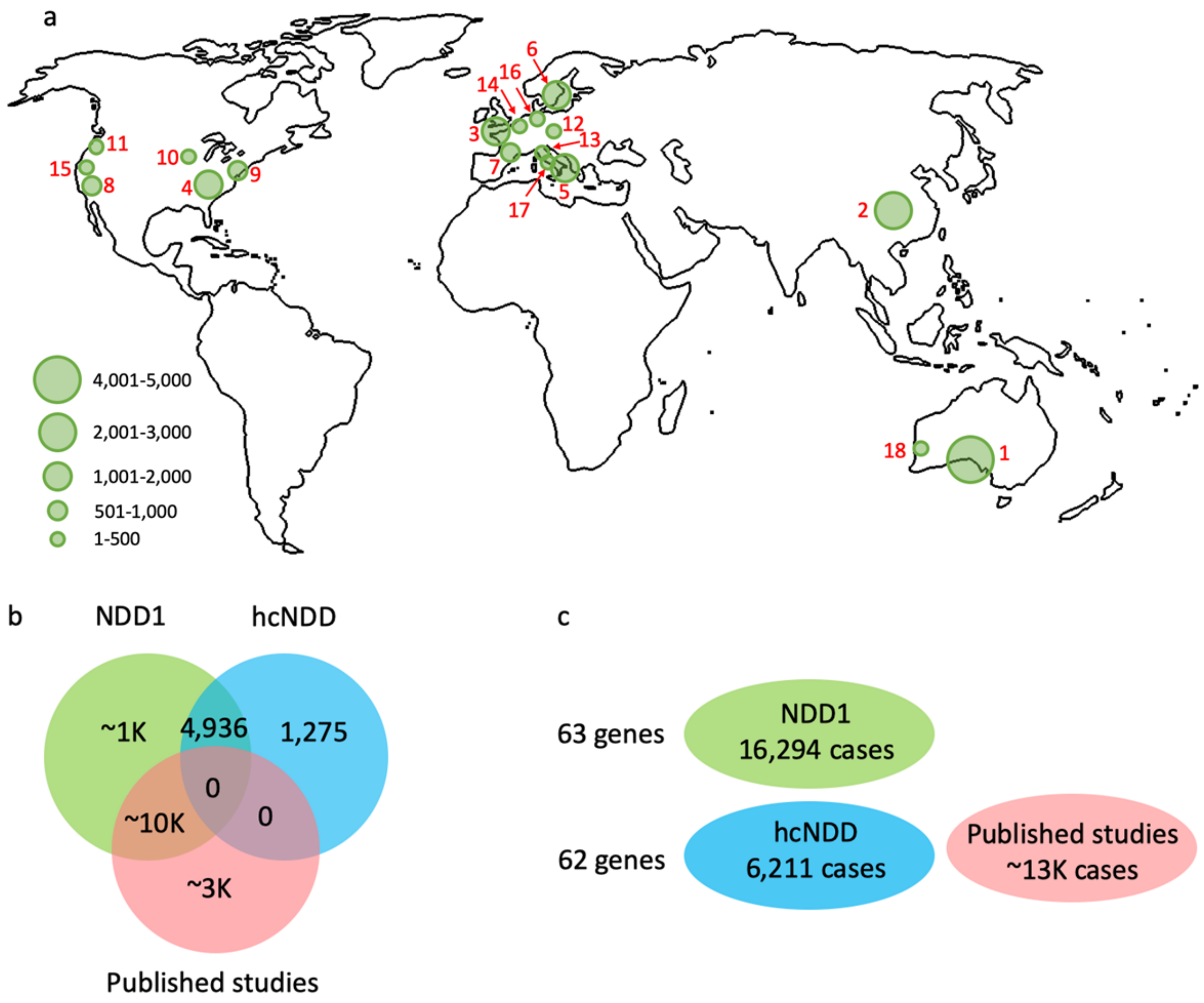

“Supplementary Fig. 1. ASID samples and smMIP targeted genes in this study. All samples sequenced in this study using smMIPs are from the ASID network. a) *Probands (n > 18K) with a primary diagnosis of ASD, DD, or ID were collected from 18 international cohorts. Circle size corresponds to the number of samples from each cohort; red numbers correspond to the cohort number in Supplementary Table 3.* ***b) The numbers (after QC) differ slightly depending on the number of genes and therefore we indicate with an approximation sign (~). Sample overlap is indicated for three designs: NDD1 (63 genes) represents a design targeting 63 genes that were not yet established as high confidence; hcNDD (62 genes) represents a design targeting genes many of which were already known; the third portion of the Venn represents previous published smMIP studies, where variants from 62 genes in hcNDD were retrieved for a combined analysis; c) the 63 genes in panel NDD1 were screened in largest number of 16,294 NDD patients, while the 62 genes in hcNDD were screened only in 6,211 NDD cases where they had not been screened before, and the same category of variants were retrieved from ~13K NDD cases (precise number of cases may different for each gene) for the same 62 genes in hcNDD.”***

We also added more details (bold text) regarding the removal of sample overlap in extended *de novo* enrichment analysis in methods section of "**Variant annotation and validation**":

"dnLGD and dnMIS variants in the de novo enrichment analysis were extracted from SPARK-27K cases with ASD (n = 6,499) from complete families and the denovo-db (v1.5) NDD subset (n = 10,927). The published exome DNMs from SPARK pilot and ASC, together with recently released exome DNMs from DDD, were also included in the extended de novo enrichment analysis with sample overlap and redundancy removed. ***For cohorts like SSC and SPARK, for which the underlying exome data are available, duplicates were identified by running the King software42, which uses identical by state (IBS) to estimate pairwise relatedness between samples. Any samples with a kinship value >0.35 were considered to be identical and counted only once. Identical samples from the same cohort were also checked for reported monozygotic twin status. We identified one pair of SSC samples and eight pairs of SPARK samples as having a kinship value >0.35. Note, samples in SPARK that overlapped with SSC samples were already removed in the final release by the SPARK Consortium. For other published cohorts, for which the underlying exome data are unavailable, the potential sample overlap identification, if applied, was described in each corresponding study. Like in the current DDD study, a total of eight duplicate samples were identified by collecting genotypes at 47 common exonic SNPs for every sample with a DNM found in another individual in the joint set; only one individual from each duplicate pair was kept with a final set of 31,058 samples analyzed. We also excluded sample overlaps reported in the literature. We excluded DD/ID samples in denovo-db (v1.5), which are also included as part of the current DDD study, and also excluded all 2,384 SSC samples in the ASC paper for potential redundancy with denovo-db (v1.5). These measures yielded a total of 48,281 NDD trios in the extended de novo enrichment analysis.***"

3. This reviewer does not know how appropriate to say exome-wide significance even in non-exome study.

The exome-wide significance here refers to the family-wise error rate (FWER) of p-value in mutational burden analysis after Bonferroni multiple test correction for approximately 20,000 genes in the human genome. This is the most stringent threshold typically applied to GWAS studies where there is no prior regarding variant class (i.e., neutral variant versus loss-of-function mutation). Inherent in FWER is a multiple test correction using the Bonferroni-corrected p-value threshold as the cutoff for significance. **Instead of referring to exome-wide significance, we now refer to either FDR or FWER significance accordingly in paper.**

4. Page 6, last line: Among 110 variants for transmission assessment, 70 inherited variants were found. How about pathogenicity of such inherited variants? Any comments are required. Or this transmission assessment was not considered in evaluation?

We went back and examined the clinical records for the 73 carrier parents for those 70 variants determined to be inherited. There are 28 carrier parents with some clinical data available, although most are fairly limited and involve very simple descriptions since

parents are not subjected to extensive phenotyping. The vast majority of the carrier parents (24/28) were classified as unaffected by clinicians. These data suggest that the variants are not necessary and sufficient to cause at least severe cognitive impairment or overt autism features, although we note that prospective studies have shown that carrier parents are more likely to carry more subtle subclinical features[1]. **We note that most of these inherited variants (50/70) were missense and/or in genes of borderline significance**. We added the following after the mentioned line to the main text:

"Transmission was successfully assessed for 110 variants, we identified 40 DNMs with 29 de novo LGD (dnLGD), 11 de novo MIS30 (dnMIS30) variants, ***and 70 inherited variants in 73 families (three inherited MIS30 variants observed in two unrelated families) with maternally inherited variants in 37 families (30 MIS30 and 7 LGD) and paternally inherited variants in 36 families (23 MIS30 and 13 LGD). The majority (50/70) of the inherited variants were missense mutations. Limited clinical data are available for 28 carrier parents (Supplementary Table 6). Among the families where the parental phenotype data is unavailable, one proband also carries a de novo missense variant (p.Arg1241Gln, CADDv1.3=15.4) in SHANK2 in addition to the paternally transmitted stop-gain variant (p.Arg860Ter) in CDK13, although the de novo variant is more likely to contribute to the proband's autism. Most of the carrier parents (24/28) were classified as unaffected with no cognitive impairment, autism, or other psychiatric problems. The remaining four carrier parents show some clinical features potentially related to the variant. One father, for example, who transmitted a MIS30 variant (p.Ser242Phe) in HNRNPR, had special education needs as he attended a school for individuals with learning disabilities but showed no obvious dysmorphic features. Similarly, a mother who transmitted a MIS30 variant (p.Arg339Gln) in CTCF showed a similar facial phenotype as the child but did not present with a clinical diagnosis of ID or ASD and was known to have attended regular school. A mother who transmitted a severe missense variant (p.Arg330Leu) in KCNQ3 was diagnosed with epilepsy but no cognitive impairment (Supplementary Table 6). Finally, one mother who transmitted a splice acceptor variant (c.1189-2A>G) in TCF12 was diagnosed with long QT syndrome and glaucoma (like the patient) but this shared feature is unlikely related to DD observed in the child or the variant in question. These findings are consistent with the idea that such transmitted variants are by themselves not necessary and sufficient to develop DD but may rather be predisposing variants with a subset of parents manifesting more subtle phenotypes[23]."***

Here, we also provided a short table (for quick reference) of the 28 inherited variants where the carrier parents with phenotype data available. We have expanded Supplementary Table 6 (column R "Inheritance_assessment") to include those clinical details.

Table 1. Phenotypic details of carrier parents.

Gene	Chr	Pos(hg19)	Ref	Alt	HGVSc	HGVSp	Var_Type	Sample(s)	Sanger_result (s)	carrier parental phenptype
KAT6B	10	76789416	C	T	c.4834C>T	p.Arg1612Cys	MIS30	ACGC_SD0049.p1	paternal inherited	Carrier parent is unaffected
KAT6B	10	76789416	C	T	c.4834C>T	p.Arg1612Cys	MIS30	ACGC_SD0306.p1	paternal inherited	Carrier parent is unaffected
WDR26	1	224606065	G	A	c.916C>T	p.Arg306Trp	MIS30	ACGC_SD0120.p1	maternal inherited	Carrier parent is unaffected
ARID2	12	46246296	C	T	c.4390C>T	p.Arg1464Cys	MIS30	ACGC_SD0201.p1	paternal inherited	Carrier parent is unaffected
ENO3	17	4860151	C	T	c.1229C>T	p.Ser410Leu	MIS30	ACGC_SD0300.p1	maternal inherited	Carrier parent is unaffected
ENO3	17	4860323	G	A	c.1313G>A	p.Arg438His	MIS30	ACGC_SD0323.p1	paternal inherited	Carrier parent is unaffected
SPEN	1	16265790	G	A	c.10864-1G>A		LGD	ACGC_SD0326.p1	paternal inherited	Carrier parent is unaffected
PHF12	17	27251092	C	T	c.622G>A	p.Asp208Asn	MIS30	ACGC_SD0339.p1	paternal inherited	Carrier parent is unaffected
HNRNPUL1	19	41811582	C	T	c.2294C>T	p.Pro765Leu	MIS30	ACGC_SD0379.p1	maternal inherited	Carrier parent is unaffected
TCF12	15	57565290	G	A	c.1808G>A	p.Arg603Gln	MIS30	AGRE_03C23149; AGRE_03C23150	paternal inherited (both)	two affected siblings in same family; carrier parent is unaffected and no autism by ADOS
SCN8A	12	52200866	C	T	c.5629C>T	p.Arg1877Trp	MIS30	AGRE_05C39269	maternal inherited	Carrier parent is unaffected and no autism by ADOS
KCNQ3	8	133198376	C	T	c.439G>A	p.Glu147Lys	MIS30	AGRE_05C48974	paternal inherited (p2 negative for this variant)	Carrier parent is unaffected and no autism by ADOS
DNM1	9	130982537	C	T	c.766C>T	p.Arg256Ter	LGD	AGRE_09C86151	paternal inherited	Carrier parent is unaffected and no autism by ADOS
TCF12	15	57535669	G	T	c.1036-1G>T	-	LGD	Antwerp_105005	maternal inherited	Carrier parent is unaffected
HNRNPR	1	23648107	G	A	c.725C>T	p.Ser242Phe	MIS30	Antwerp_80796	paternal inherited	**Carrier father had special education needs (attented school for people with borderline intelligence), but he showed no dysmorphic features.**
CLTC	17	57737944	C	T	c.1165C>T	p.Pro389Ser	MIS30	Leiden_D1.06.08940	paternal inherited	Carrier parent no developmental delay or autism
ENO3	17	4859960	G	A	c.1187G>A	p.Gly396Glu	MIS30	Leiden_D1.12.04351	paternal inherited	Carrier parent no developmental delay or autism
TCF12	15	57525037	G	A	c.953G>A	p.Gly318Glu	MIS30	Leiden_D1.12.09041	paternal inherited	Carrier parent no developmental delay or autism
SPEN	1	16199609	C	T	c.382C>T	p.Arg128Cys	MIS30	Leiden_D1.12.12474	paternal inherited	Carrier parent no developmental delay or autism
BRPF1	3	9781575	C	T	c.1492C>T	p.Arg498Trp	MIS30	Leiden_D1.12.15391	maternal inherited	Carrier parent no developmental delay or autism
BRPF1	3	9782554	C	T	c.1651C>T	p.Arg551Trp	MIS30	Leiden_D2.09.10079	maternal inherited	Carrier parent no developmental delay or autism
KCNQ3	8	133192474	C	T	c.707G>A	p.Arg236His	MIS30	Leuven_350183	paternal inherited	Carrier parent no cognitive impairment, autism or psychiatric problems
TCF12	15	57544617	A	G	c.1189-2A>G	-	LGD	Leuven2_84254374	maternal inherited	**Carrier mother has long QT syndrome and glaucoma (so as the patient), but she has no autism or other psychiatric problems.**
CTCF	16	67650711	G	A	c.1016G>A	p.Arg339Gln	MIS30	Swedish_1209-11D	maternal inherited	**Variant assessed as a VUS. The phenotype of the carrier mother is unspecific; she has the same facial phenotype as the child, but she does not have a known diagnosis of ID and/or ASD. In addition, she went to regular school.**
CTCF	16	67671596	G	A	c.2000-1G>A		LGD	TASC_211-5234-3	paternal inherited	Carrier parent is unaffected
TCF12	15	57535733	C	T	c.1099C>T	p.Pro367Ser	MIS30	TASC_217-14129-2250	maternal inherited	Carrier parent is unaffected
KCNQ3	8	133186541	C	A	c.989G>T	p.Arg330Leu	MIS30	Troina2_3664-6681	maternal inherited	**The mother is epileptic, but no more.**
SPEN	1	16262237	C	T	c.9502C>T	p.Arg3168Ter	LGD	Troina3_2017_04921_12375	paternal inherited	Carrier parent is unaffected

5. In Figure 3, which variants are novel?

Variants listed above the protein diagram are new to this study and previously unpublished, while the ones below were published previously. We split the former Figure 3 into updated Figure 3 and Figure 4 and also added brackets indicating variants above and below, like in (a), and have stated this in the legend to make it clearer.

Updated Figure 3:

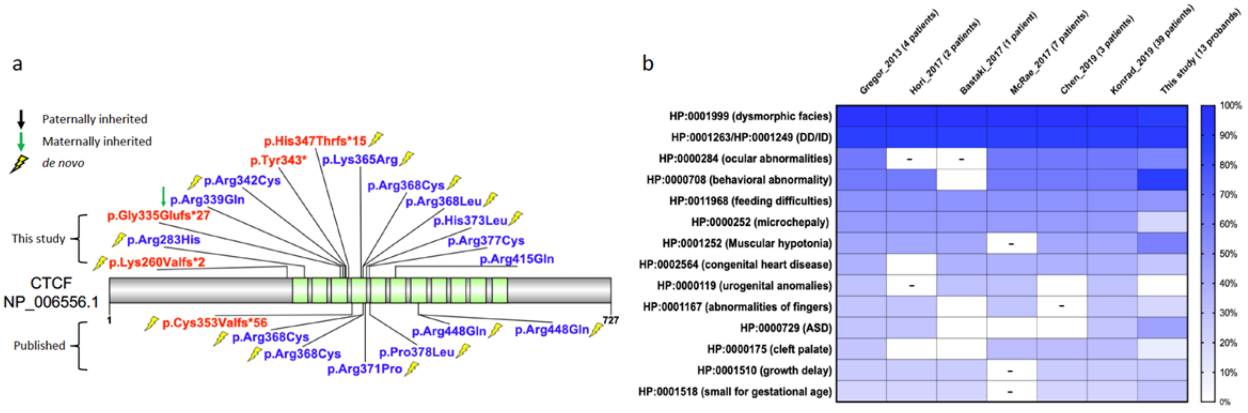

*“**Figure 3. Distribution of severe patient variants and the genotype–phenotype correlations in CTCF.** (**a**) LGD (red) and MIS30 (blue) variants are depicted against a protein model for CTCF. **Variants new to this study are shown above the protein while published DNMs from denovo-db (v1.5) are below.** Variants are flagged with yellow lightning bolt if is de novo. Annotated protein domains are shown (colored blocks) for the largest protein isoforms. (**b**) Heatmap depicts the common clinical features for patients carrying CTCF severe variants by using the specific HPO annotation (rows), which were retrieved from published studies and our cohort (columns). Phenotypic enrichment is shown according to the features’ recurrence labeled by the increment of color degree. The items with no data available were labeled with “-” and were excluded in the frequency analysis.”*

Updated Figure 4:

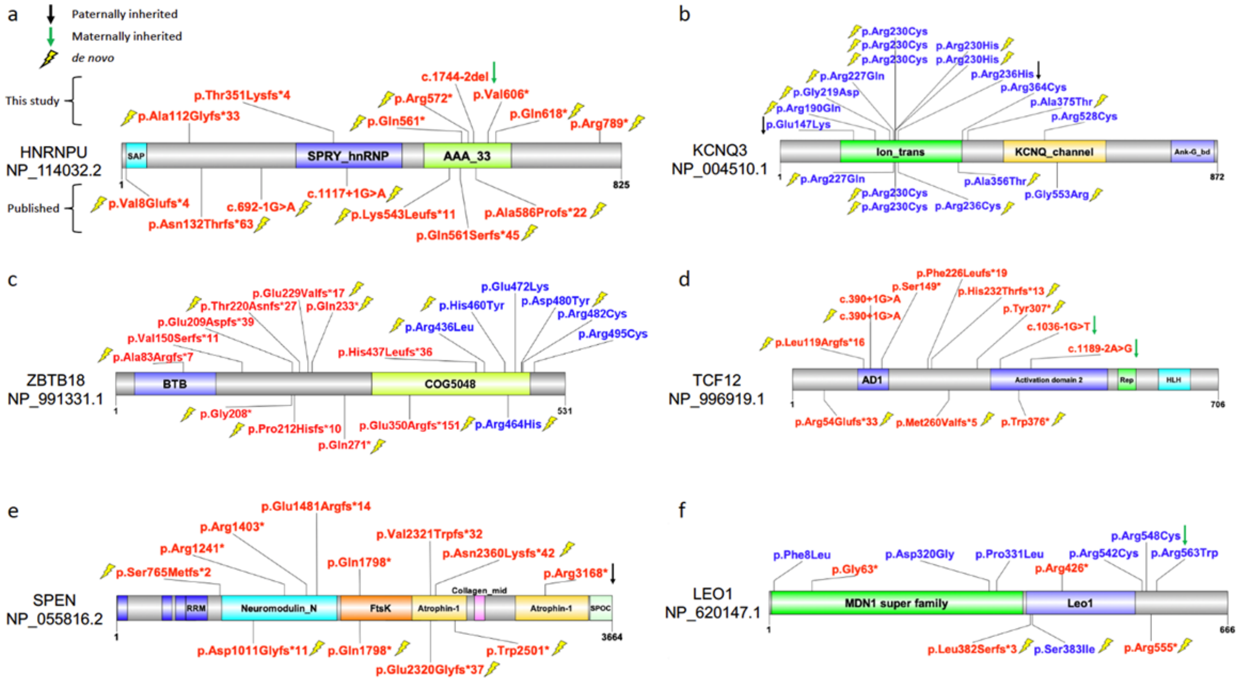

*“**Figure 4. Distribution of severe patient variants in six genes.** Protein diagrams are shown for HNRNPU (**a**), KCNQ3 (**b**), ZBTB18 (**c**), TCF12 (**d**), SPEN (**e**), and LEO1(**f**) with the same displaying metrics that applied in Figure 3. Validated LGD (red) and MIS30 (blue) variants are plotted. **Variants listed above the protein model are new to this study, while the ones below were published previously.** Paternal (green arrow) and maternal (black arrow) inheritance are shown if determined. A yellow lightning bolt denotes a de novo mutation.”*

6. Page 8, line 18: In reevaluation of genes for excess DNMs section, this reviewer cannot fully understand “6,499 new ASD trios from 5,911 complete families”.

This difference is due to the fact that there are both simplex and multiplex families under consideration. Among the 5,911 complete families, there are 1,150 multiplex families (including 1,738 ASD patients) with more than one affected individual in each family. To avoid confusion, we updated the sentence as below:

*“we identified 99 dnLGD and 104 dnMIS (including 31 dnMIS30) variants in 6,499 new ASD **patients** from 5,911 complete families **(4,761 simplex and 1,150 multiplex families)** in our recent analysis of 27,270 SPARK exomes”*

7. Page 10, line 21: The authors performed case-control analysis of ultra rare and damaging variants at 125 candidate genes and found that 48 genes were significantly enriched (5% FDR). The authors stated that “SPEN is newly identified in this study with a significant burden” at the section of “Genotype-phenotype correlations” while the authors didn’t mention which other 47 genes were novel or not. The authors should describe what in the 47 genes were novel.

There are 25 genes that we regard as now newly reaching mutation burden significance for ultra-rare LGD and/or MIS30 among the 48 genes (5% FDR) in this study. We mention this in abstract, also indicate in Table 1 in paper (genes with *) and Supplementary Table 11. To make it clearer, we further added the following sentence (in bold) at the end of section “**Genes with an excess burden of ultra-rare severe variants.**”

“We identified 48 genes with a significant excess of LGD and/or MIS30 ($q_{mutBurden}$ < 0.05, corrected n_{genes} = 125, variant count > 1) (Table 1, Figure 2, Supplementary Table 11) in cases. Of these, six genes (ADNP, CHD8, DYRK1A, GRIN2B, POGZ, and SCN2A) also reached a more stringent ***significance*** *threshold* ***that pass*** *exome-wide* **Bonferroni correction at the family-wise error rate** ***(FWER)*** *for LGD variants ($p_{mutBurden}$ < 1.25E-06, corrected n_{genes} = 20,000, variant count > 1).* ***Among the 48 significant genes, we identified 25 genes that show evidence of ultra-rare LGD and/or MIS30 (FDR 5%) burden for the first time in this large-scale case–control study, although 21 of these have been shown previously to show enrichment for DNMs (Supplementary Table 11).****”*

And here we also provided a short table 2 for quick reference for the 25 gene newly showing mutation burden significance in this study. We indicated this in Supplementary Table 11. Coe253 (Coe *et al*., 2019[2]), ASC102 (Satterstrom *et al*., 2020[3]), and DDD299 (Kaplanis et al., 2019, bioRxiv preprint) indicate three list of genes that previously reported with DNM significance.

Table 2. Genes newly showing mutation burden significance.

Gene	New mutation burden significance (FDR 5%)	Reported DNM significance		
		Coe253	ASC102	DDD299
CHD2	LGD	Yes	Yes	Yes
SETBP1	LGD	Yes		Yes
WAC	LGD	Yes	Yes	Yes
ASXL3	LGD	Yes	Yes	Yes
MYT1L	LGD	Yes	Yes	Yes
BRAF	MIS30	Yes		Yes
CHAMP1	LGD	Yes		Yes
HNRNPU	LGD	Yes		Yes
KAT6A	LGD	Yes		Yes
NEXMIF	LGD	Yes		Yes
SATB2	LGD	Yes		Yes
ZBTB18	LGD	Yes		Yes
PHIP	LGD	Yes		Yes
ZNF292	LGD	Yes		Yes
BRPF1	LGD	Yes		Yes
PHF12	LGD	Yes	Yes	Yes
SPEN	LGD	Yes		Yes
TBR1	LGD	Yes	Yes	
TCF12	LGD	Yes		
KMT2E	LGD		Yes	Yes
ZMYM2	LGD			Yes
RELN	LGD			
AHNAK	LGD			
PHF7	LGD			
PASK	LGD			

Reviewer #2 (Remarks to the Author):

The authors present data on 125 candidate de novo mutations genes for over 16,000 subjects with NDD. The authors make an excellent point in the Introduction, that, to date, very few of the reported candidate genes have been studied at sufficient depth with sufficient cases to provide genome-wide statistical significance or to establish meaningful genotype-phenotype correlations. **The approach outlined here goes some distance to provide that for a subset of implicated genes, and hopefully will set precedents for the study of further candidate genes.** Where exome/genome sequence can be used as the discovery tool, the approach here attempts to validate these discoveries in a meaningful way. **The paper is very well written, and a valuable contribution and reference point for the research and clinical communities.**

I have a few comments and suggestions for clarification:

What criteria or other considerations were used to rank and select the 125 candidate genes chosen?

There were two sets of genes: new candidates (NDD1) and high-confidence genes (hcNDD). We now elaborate upon the criteria for gene selection in the methods (“Candidate genes” section) for each group. Supplementary Table 1 also provides a complete list of genes and the selection criteria. We updated the description (in bold) to make it more legible.

*“**Candidate genes. We considered two sets of genes: new candidates (NDD1) for investigation and high-confidence genes (hcNDD) that have been previously implicated in NDDs. Different criteria were used in selecting these two groups. NDD1.** We ranked and selected candidate genes **for which no smMIP sequencing had been performed previously. We initially ranked all genes based** on the DNMs from published NDD trios cataloged in denovo-db (v1.5), but excluding the following: genes associated with well-known syndromes based on OMIM, genes with extremely high-GC **content**, and genes with high counts of LGD and MIS30 variants in the ExAC non-psych controls. In total, 65 genes were selected for screening with: i) 43 genes showing excess DNM[25]; ii) 14 genes with evidence of autism sex bias[39]; iii) six genes from a network analysis of high-functioning autism indicated previously; iv) and two genes (H2AC6 and H1-4) that were considered within a CNV candidate. **hcNDD.** We continually reselected 62 top candidate genes from our previous smMIP panels[3], **mainly ranked by the reported number of DNMs from the published NDD trios in denovo-db (v1.5) and number of ultra-rare severe LGD and MIS30 variants identified in targeted sequencing of >13,000 NDD cases. We sequenced an additional 6,666 newly recruited NDD cases that had not been previously sequenced using smMIPs. These served as positive controls of known disease genes in this study allowing for the discovery of additional cases for phenotypic evaluation. During the selection of these 125 genes, we evaluated the success rate of all smMIPs for each gene as part of our optimization experiments. We excluded genes, for example, where >20% of smMIPs failed to provide sufficient coverage even after 50-fold spike-in. We also balanced the total number of smMIPs per gene in each panel needed to achieve sufficient sequence depth. In particular, large genes requiring more than 200 smMIPs were triaged to allow a greater number of more moderate-sized genes to be considered.** Supplementary Table 1 **lists the** genes with **detailed** selection criteria.”*

Was it a trio study or just probands? It implies in the Results section that parents were tested (by referring to paternal/maternal/de novo) but I cannot find any explicit mention of this in the methods. Presumably Sanger sequencing, but this should be clearer.

Targeted sequencing and mutation burden analysis were performed initially on probands, but once potentially pathogenic mutations were identified, a follow-up was attempted in the context of the family wherever DNA was available. Thus, smMIP sequencing was applied to probands and after Sanger validation confirmed the variant we assessed parental DNA. It should be pointed out, however, that the *de novo* enrichment analysis to rank order the significance of genes was established based on parent–child trio information. Details are described in the corresponding methods section. To clarify, we further updated (in bold) the Figure 1 legend:

*"**Figure 1. Overview of study design. Targeted sequencing was performed in probands for two gene panels: NDD1 (63 genes) and hcNDD (62 genes).** The same categories of variants were retrieved from three previously published smMIP studies for 62 hcNDD genes. All smMIP variants were combined; redundant samples were eliminated and compared to the same category of variants from ExAC non-psych controls. The number of variants is after the exclusion of false positive variants and variants with insufficient coverage in ExAC. Mutation burden analysis identified 48 FDR significant genes ($q_{mutBurden}$ < 0.05, Benjamini–Hochberg correction for 125 genes), of which six reached FWER significance ($p_{mutBurden}$ < 1.25E-06, Bonferroni correction for 20,000 genes and two tests); **DNMs of the 125 genes used in this study were identified from exome sequencing in 10,927 published NDD trios and 6,499 new ASD trios that combined as 17,426 NDD parent-child trios.** A separate de novo enrichment analysis, using two statistical models (CH model and denovolyzeR), identified 90 FDR significant genes ($q_{dnEnrich}$ < 0.05, Benjamini–Hochberg correction for 18,946 genes in CH model and 19,618 genes in denovolyzeR), of which, 61 genes reach FWER significance ($p_{dnEnrich}$ < 3.64E-07, Bonferroni correction for 19,618 genes and seven tests) for excess DNM. There is a significant overlap (40 genes) of the significant genes suggested by the two approaches. Then we performed genotype-phenotype correlation analysis for seven NDD risk genes (CTCF, HNRNPU, KCNQ3, ZBTB18, TCF12, SPEN, and LEO1) and presented a clearer clinical picture of each gene."*

Minor comments:
Page 7 line 13: delete "are"

Deleted.

Page 9: paragraph 2, line2-3: insert "were" after "from families that.."

Added.

Figure 3: I'm concerned that Figs 3c-h include text indicating specific mutations that will be too small to read (especially Fig3g, SPEN which is an even smaller font). I would suggest that these figures be separated from the rest of Fig 3 and moved into a new figure where they can be enlarged.

Thanks for this suggestion. In our revisions, the former Figure 3a and b are now split from the previous Figure 3c-h, which have moved to Figure 4. We also updated the font, especially in previous Figure 3g to make it more legible.

Updated Figure 3:

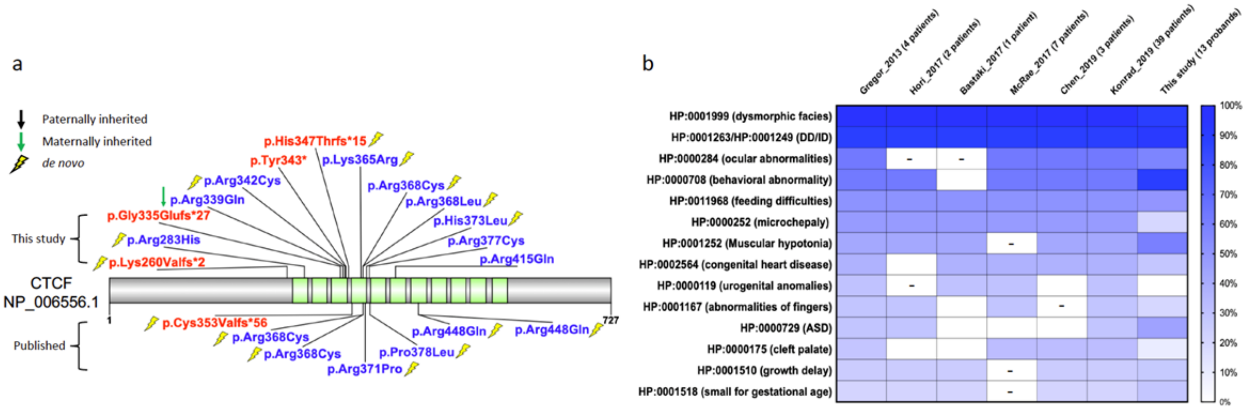

Updated Figure 4:

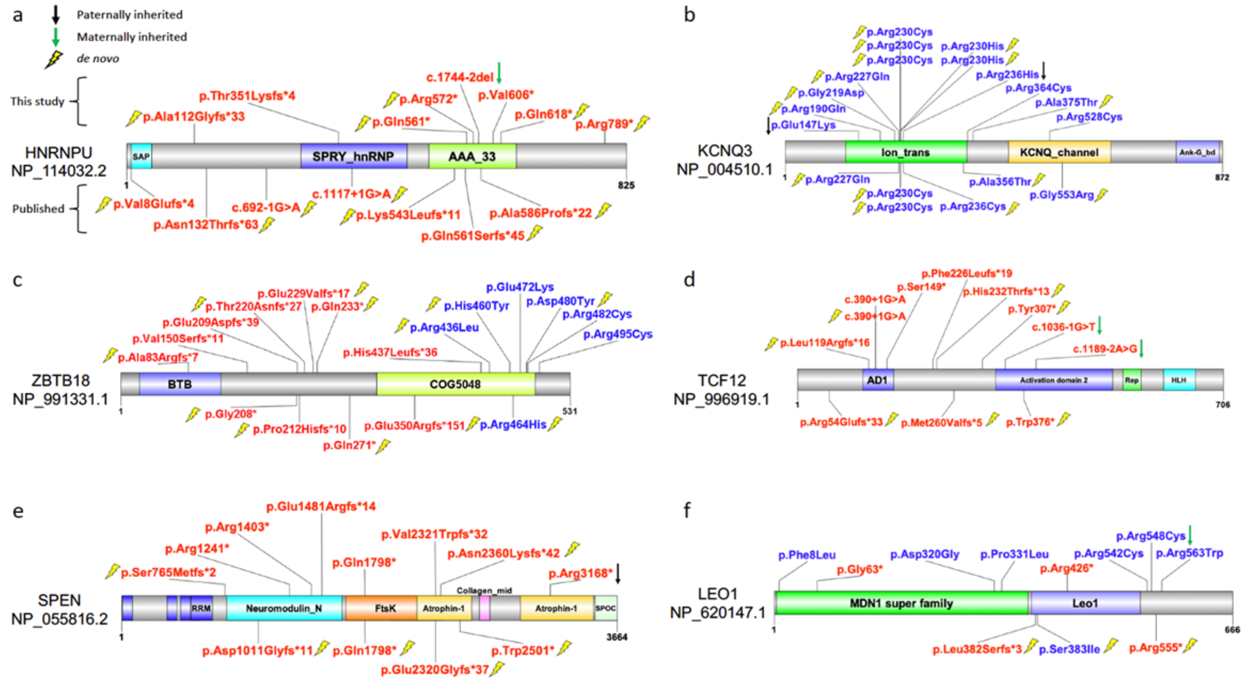

References

1. Guo, H. *et al.* Disruptive mutations in TANC2 define a neurodevelopmental syndrome associated with psychiatric disorders. *Nat Commun* **10**, 4679 (2019).
2. Coe, B.P. *et al.* Neurodevelopmental disease genes implicated by de novo mutation and copy number variation morbidity. *Nature Genetics* **51**, 106-116 (2019).
3. Satterstrom, F.K. *et al.* Large-Scale Exome Sequencing Study Implicates Both Developmental and Functional Changes in the Neurobiology of Autism. *Cell* **180**, 568-584 e23 (2020).

REVIEWERS' COMMENTS:

Reviewer #1 (Remarks to the Author):

This reviewer thinks most of genes were already recognized as disease-causative, but, in the statistical point of view, authors are successful in demonstrating “novel” (newly reaching statistically significant) genes using a large data set (Tables 1, 2, S11). As a few genes like PHF12, PHF7 and LEO1 are not fully established yet, information of their variants are useful. They showed a much clearer structure of analyzed populations in the revised manuscript than those in the previous manuscript (still complicated, though). “Exome-wide” was not used and now changed to FWER. The 70 inherited variants are properly described and explained. Figures 3 and 4 are updated in showing novel and reported variants as well as de novo variants. The 25 genes with the mutation burden significance (FDR 5%) are shown in table 2 and supplementary table 11. Thus, this reviewer thinks authors addressed most of them.

Reviewer #2 (Remarks to the Author):

The authors have revised the manuscript, which is now acceptable for publication, in my view.

Point-by-point referee response
Wang et al.

REVIEWERS' COMMENTS:

Reviewer #1 (Remarks to the Author):

This reviewer thinks most of genes were already recognized as disease-causative, but, in the statistical point of view, authors are successful in demonstrating “novel” (newly reaching statistically significant) genes using a large data set (Tables 1, 2, S11). As a few genes like PHF12, PHF7 and LEO1 are not fully established yet, information of their variants are useful. They showed a much clearer structure of analyzed populations in the revised manuscript than those in the previous manuscript (still complicated, though). “Exome-wide” was not used and now changed to FWER. The 70 inherited variants are properly described and explained. Figures 3 and 4 are updated in showing novel and reported variants as well as de novo variants. The 25 genes with the mutation burden significance (FDR 5%) are shown in table 2 and supplementary table 11. Thus, this reviewer thinks authors addressed most of them.

Thanks!

Reviewer #2 (Remarks to the Author):

The authors have revised the manuscript, which is now acceptable for publication, in my view.

Thanks!
